# The visual mismatch negativity elicited with visual speech stimuli

**DOI:** 10.3389/fnhum.2013.00371

**Published:** 2013-07-16

**Authors:** Benjamin T. Files, Edward T. Auer, Lynne E. Bernstein

**Affiliations:** ^1^Neuroscience Graduate Program, University of Southern CaliforniaLos Angeles, CA, USA; ^2^Communication Neuroscience Laboratory, Department of Speech and Hearing Science, George Washington UniversityWashington, DC, USA

**Keywords:** speech perception, visual perception, lipreading, scalp electrophysiology, mismatch negativity (MMN), hemispheric laterazation for speech

## Abstract

The visual mismatch negativity (vMMN), deriving from the brain's response to stimulus deviance, is thought to be generated by the cortex that represents the stimulus. The vMMN response to visual speech stimuli was used in a study of the lateralization of visual speech processing. Previous research suggested that the right posterior temporal cortex has specialization for processing simple non-speech face gestures, and the left posterior temporal cortex has specialization for processing visual speech gestures. Here, visual speech consonant-vowel (CV) stimuli with controlled perceptual dissimilarities were presented in an electroencephalography (EEG) vMMN paradigm. The vMMNs were obtained using the comparison of event-related potentials (ERPs) for separate CVs in their roles as deviant vs. their roles as standard. Four separate vMMN contrasts were tested, two with the perceptually *far* deviants (i.e., “zha” or “fa”) and two with the *near* deviants (i.e., “zha” or “ta”). Only *far* deviants evoked the vMMN response over the left posterior temporal cortex. All four deviants evoked vMMNs over the right posterior temporal cortex. The results are interpreted as evidence that the left posterior temporal cortex represents speech contrasts that are perceived as different consonants, and the right posterior temporal cortex represents face gestures that may not be perceived as different CVs.

## Introduction

The visual mismatch negativity (vMMN) paradigm was used here to investigate visual speech processing. The MMN response was originally discovered and then extensively investigated with auditory stimuli (Näätänen et al., 1978, 2011). The classical auditory MMN is generated by the brain's automatic response to a change in repeated stimulation that exceeds a threshold corresponding approximately to the behavioral discrimination threshold. It is elicited by violations of regularities in a sequence of stimuli, whether the stimuli are attended or not, and the response typically peaks 100–200 ms after onset of the deviance (Näätänen et al., 1978, 2005, 2007). The violations that generate the auditory MMN can range from low-level stimulus deviations such as the duration of sound clicks (Ponton et al., 1997) to high-level deviations such as speech phoneme category (Dahaene-Lambertz, 1997). More recently, the vMMN was confirmed (Pazo-Alvarez et al., 2003; Czigler, 2007; Kimura et al., 2011; Winkler and Czigler, 2012). It too is elicited by a change in regularities in a sequence of stimuli, across different levels of representation, including deviations caused by spatiotemporal visual features (Pazo-Alvarez et al., 2004), conjunctions of visual features (Winkler et al., 2005), emotional faces (Li et al., 2012; Stefanics et al., 2012), and abstract visual stimulus properties such as bilateral symmetry (Kecskes-Kovacs et al., 2013) and sequential visual stimulus probability (Stefanics et al., 2011).

Speech can be perceived visually by lipreading, and visual speech perception is carried out automatically by hearing as well as by hearing-impaired individuals (Bernstein et al., 2000; Auer and Bernstein, 2007). Inasmuch as perceivers can visually recognize the phonemes (consonants and vowels) of speech through lipreading, the stimuli are expected to undergo hierarchical visual processing from simple features to complex representations along the visual pathway (Grill-Spector et al., 2001; Jiang et al., 2007b), just as are other visual objects, including faces (Grill-Spector et al., 2001), facial expression (Li et al., 2012; Stefanics et al., 2012), and non-speech face gestures (Puce et al., 1998, 2000, 2007; Bernstein et al., 2011). Crucially, because the vMMN deviation detection response is thought to be generated by the cortex that represents the standard and deviant stimuli (Winkler and Czigler, 2012), it should be possible to obtain the vMMN in response to deviations in visual speech stimuli. However, previous studies in which a speech vMMN was sought produced mixed success in obtaining a deviance response attributable to visual speech stimulus deviance detection (Colin et al., 2002, 2004; Saint-Amour et al., 2007; Ponton et al., 2009; Winkler and Czigler, 2012). A few studies have even sought an auditory MMN in response to visual speech stimuli (e.g., Sams et al., 1991; Möttönen et al., 2002).

The present study took into account how visual stimuli conveying speech information might be represented and mapped to higher levels of cortical processing, say for speech category perception or for other functions such as emotion, social, or gaze perception. That is, the study was specifically focused on the perception of the physical visual speech stimulus. The distinction between representations of the forms of exogenous stimuli vs. representation of linguistic categories is captured in linguistics by the terms *phonetic form* vs. *phonemic category*. Phonetic forms are the exogenous physical stimuli that convey the linguistically-relevant information used to perceive the speech category to which the stimulus belongs. Visual speech stimuli convey linguistic phonetic information primarily via the visible gestures of the lips, jaw, cheeks, and tongue, which support the system of phonological contrasts that underly speech phonemes (Yehia et al., 1998; Jiang et al., 2002; Bernstein, 2012). Phonemic categories are the consonant and vowel categories that a language uses to differentiate and represent words. If visual speech is processed similarly to auditory speech stimuli, functions related to higher-level language processing, such as categorization and semantic associations, are carried out beyond the level of exogenous stimulus form representations (Scott and Johnsrude, 2003; Hickok and Poeppel, 2007).

This study was concerned with the implications for cortical representation of visual speech stimuli in the case that speech perception is generally left-lateralized. There is evidence for form-based speech representations in high-level visual areas, and there is evidence that they are left-lateralized (Campbell et al., 2001; Bernstein et al., 2011; Campbell, 2011; Nath and Beauchamp, 2012). For example, Campbell et al. (1986) showed that a patient with right-hemisphere posterior cortical damage failed to recognize faces but had preserved speech lip-shape recognition, and that a patient with left-hemisphere posterior cortical damage failed to recognize speech lip-shapes but had preserved face recognition.

Recently, evidence for hemispheric lateralization was obtained in a study designed to investigate specifically the site/s of specialized visual speech processing. Bernstein et al. (2011), applied a functional magnetic resonance imaging (fMRI) block design while participants viewed video and point-light speech and non-speech stimuli and tiled control stimuli. Participants were imaged during localizer scans for three regions of interest (ROIs), the fusiform face area (FFA) (Kanwisher et al., 1997), the lateral occipital complex (LOC) (Grill-Spector et al., 2001), and the human visual motion area V5/MT. These three areas were all under-activated by speech stimuli. Although both posterior temporal cortices responded to speech and non-speech stimuli, only in the left hemisphere was an area found with differential sensitivity to speech vs. non-speech face gestures. It was named the *temporal visual speech area* (TVSA) and was localized to the posterior superior temporal sulcus and adjacent posterior middle temporal gyrus (pSTS/pMTG), anterior to cortex that was activated by non-speech face movement in video and point-light stimuli. TVSA is similarly active across video and point-light stimuli. In contrast, right-hemisphere activity in the pSTS was not reliably different for speech vs. non-speech face gestures. Research aimed at non-speech face gesture processing has also produced evidence of right-hemisphere dominance for non-speech face gestures, with a focus in the pSTS (Puce et al., 2000, 2003).

**Figure 1:**
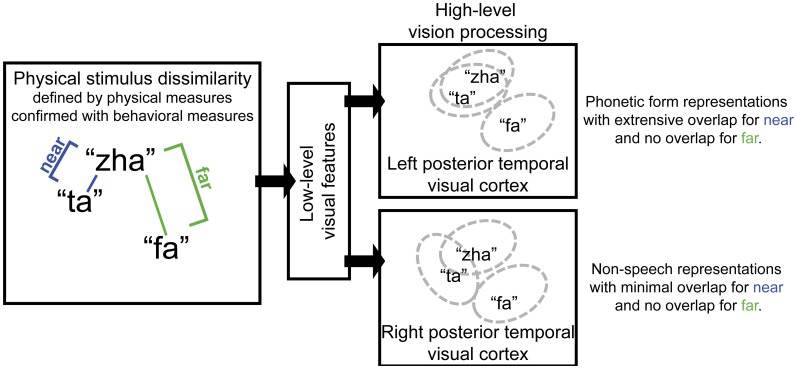
**Schematic diagram of the proposed roles for left and right posterior temporal cortices in visual speech perception**. Left posterior temporal visual cortex is hypothesized to represent phonetic forms that support eventual phoneme categorization and to therefore be invariant to variation in facial motion that is unlikely to reliably support speech perception. Pairs of visual speech syllables are predicted to activate largely overlapping populations of neurons in left posterior temporal cortex when the syllables in the pair are perceptually similar (i.e., they are perceptually *near*). But non-overlapping populations of neurons in left posterior temporal cortex represent syllables that are perceptually highly dissimilar (i.e., they are perceptually *far*). In contrast, the right posterior temporal cortex is hypothesized to represent non-speech facial gestures. Near pairs of visual speech syllables are predicted to activate only partially overlapping populations of neurons in right posterior temporal visual cortex, and far pairs are predicted to activate non-overlapping populations.

The approach in the current study was based on predictions for how the representation of visual speech stimuli should differ for the right vs. left posterior temporal cortex under the hypothesis that the left cortex has tuning for speech, but the right cortex has tuning for non-speech face gestures. Specifically, lipreading relies on highly discriminable visual speech differences. Visual speech phonemes are not necessarily as distinctive as auditory speech phonemes. Visual speech consonants are known to vary in terms of how distinct they are from each other, because some of the distinctive speech features used by listeners (e.g., voicing, manner, nasality, place) to distinguish phonemes are not visible or are less visible to lipreaders (Auer and Bernstein, 1997; Bernstein, 2012). A left posterior temporal cortex area specialized for speech processing, part of an extensive speech processing pathway, is expected to be tuned to represent linguistically useful exogenous phonetic forms, that is, forms that can be mapped to higher-level linguistic categories, such as phonemes. However, when spoken syllables (e.g., “zha” and “ta”) do not provide enough visual phonetic feature information, their representations are expected to generalize. That is, the indistinct stimuli activate overlapping neural populations. This is depicted in Figure 1, for which the visually *near* (perceptual categories are not distinct) syllables “ta” and “zha” are represented by almost completely overlapping ovals in the box labeled *left posterior temporal visual cortex*. The perceptually far stimulus “fa,” a stimulus that shares few visible phonetic features with “zha,” is depicted within its own non-overlapping oval in that box. Here, using the vMMN paradigm, a deviance response was predicted for the left hemisphere with the stimuli “zha” vs. “fa,” representing a *far* contrast. But the *near* contrast “zha”-“ta,” depicted in Figure 1, was not predicted to elicit the vMMN response by the left posterior temporal cortex for “zha” or for “ta” syllables.

In contrast, the right posterior temporal cortex, with its possible dominance for processing simple non-speech face motions such as eye open vs. closed, and simple lips open vs. closed (Puce et al., 2000, 2003), was predicted to generate a deviance response to both perceptually *near* and *far* speech stimulus contrasts. The depiction in Figure 1 for the right posterior temporal cortex shows that the stimulus differences are represented there more faithfully (i.e., there are more neural units that are not in common). The right posterior temporal cortex is theoretically more concerned with perception of non-speech face gestures, for example, gestures related to visible emotion or affect: The representations may even be more analog in the sense that they are not used as input to a generative system that relies on combinations of representations (i.e., vowels and consonants) to produce a very large vocabulary of distinct words.

Even very simple low-level visual features or non-speech face or eye motion in the speech video clips can elicit the vMMN (Puce et al., 2000, 2003; Miki et al., 2004; Thompson et al., 2007). With natural speech production, phonetic forms vary from one production to the next. An additional contribution to variability is the virtually inevitable shifts in the talker's head position, eye gaze, eyebrows, etc., from video recording to recording. Subtle differences are not necessarily so obvious on a single viewing, but the vMMN paradigm involves multiple stimulus repetitions, which can render subtle differences highly salient.

The approach here was to use two recordings for each consonant and to manipulate the stimuli to minimize non-phonetic visual cues that might differentiate the stimuli. The study design took into account the likelihood that the deviance response to speech stimuli would be confounded with low-level stimulus differences, if it involved a stimulus as standard (e.g., “zha”) vs. a different stimulus as deviant (e.g., “fa”). Therefore, the vMMN was sought using the event-related potentials (ERPs) obtained with the same stimulus (e.g., “zha”) in its two possible roles of standard and deviant. Stimulus discriminability was verified prior to ERP recording. During ERP recording, participants monitored for a rare target phoneme to engage their attention and hold it at the level of phoneme categorization, rather than at the level of stimulus discrimination.

## Method

### Participants

Participants were screened for right-handedness (Oldfield, 1971), normal or corrected to normal vision (20/30 or better in both eyes using a traditional Snellen chart), normal hearing, American English as a first and native language, and no known neurological deficits. Lipreading was assessed with a screening test that has been used to test a very large sample of normal hearing individuals (Auer and Bernstein, 2007). The screening cutoff was 15% words correct in isolated sentences to assure that participants who entered the EEG experiment had some lipreading ability. Forty-nine individuals were screened (mean age = 23 years), and 24 (mean age = 24, range 21–31, 18 female, lipreading score *M* = 28.7% words correct) met the inclusion criteria for entering the EEG experiment. The EEG data from 11 participants (mean age = 23.2, range 19–31, 7 female, lipreading score *M* = 33.0) were used here: One participant was lost to contact, one ended the experiment early, two had unacceptably high initial impedance levels and were not recorded, and nine had high electrode impedances, excessive bridging between electrodes, or unacceptable noise levels. Informed consent was obtained from all participants. Participants were paid. The research was approved by the Institutional Review Boards at George Washington University and at the University of Southern California.

### Stimuli

#### Stimulus dissimilarity

The stimuli for this study were selected to be of predicted perceptual and physical dissimilarities. Estimates of the dissimilarities and the video speech stimuli themselves were obtained from Jiang et al. (2007a), which gives a detailed description of the methods for predicting and testing dissimilarity. Based on the dissimilarity measures in Jiang et al. (2007a), the stimulus pair “zha”—“fa,” with modeled dissimilarity of 4.04, was chosen to be perceptually *far*, and the stimulus pair “zha”—“ta,” with modeled dissimilarity of 2.28 was chosen to be perceptually *near*. In a subsequent study, Files and Bernstein (submitted) tested whether the modeled dissimilarities among a relatively large selection of syllables correctly predicted stimulus discriminability, and they did.

#### Stimulus video

Stimuli were recorded so that the talker's face filled the video screen, and lighting was from both sides and slightly below his face. A production quality camera (Sony DXC-D30 digital) and video recorder (Sony UVW 1800) were used simultaneously with an infrared motion capture system (Qualisys MCU120/240 Hz CCD Imager) for recording 3-dimensional (3D) motion of 20 retro-reflectors affixed to the talker's face. The 3D motion recording was used by Jiang et al. (2007a) in developing the dissimilarity estimates. There were two video recordings of each of the syllables, “zha,” “ta,” and “fa” that were used for eliciting the vMMNs. Two tokens of “ha,” and of “va” were used as targets to control attention during the vMMN paradigm. All video was converted to grayscale.

In order to reduce differences in the durations of preparatory mouth motion across stimulus tokens and increase the rate of data collection, some video frames were removed from slow uninformative mouth opening gestures. But most of the duration differences were reduced by removing frames from the final mouth closure. No frames were removed between the sharp initiation of articulatory motion and the quasi-steady-state portion of the vowel.

During the EEG experiment, the video clips were displayed contiguously through time. To avoid responses due to minor variations in the position of the head from the end of one token to the beginning of the next, morphs of 267 ms were generated (Abrosoft's FantaMorph5) to create smooth transitions from one token to the next. The morphing period corresponded to the inter-stimulus-interval.

The first frame of each token was centered on the video monitor so that a motion-capture dot that was affixed at the center of the upper lip was at the same position for each stimulus. Also, stimuli were processed so that they would not be identifiable based solely on the talker's head movement. This was done by adding a small amount of smooth translational motion and rotation to each stimulus on a frame-by-frame basis. The average motion speed was 0.5 pixels per frame (0.87° of visual angle/s), with a maximum of 1.42 pixels per frame (2.5°/s). Rotation varied between plus and minus 1.2° of tilt, with an average change of 0.055° of tilt per frame (3.28°/s) and a maximum change of 0.15° of tilt per frame (9.4° of tilt/s). A stationary circular mask with radius 5.5° of visual angle and luminance equal to the background masked off the area around the face of the talker.

#### Stimulus alignment and deviation points

**Figure 2:**
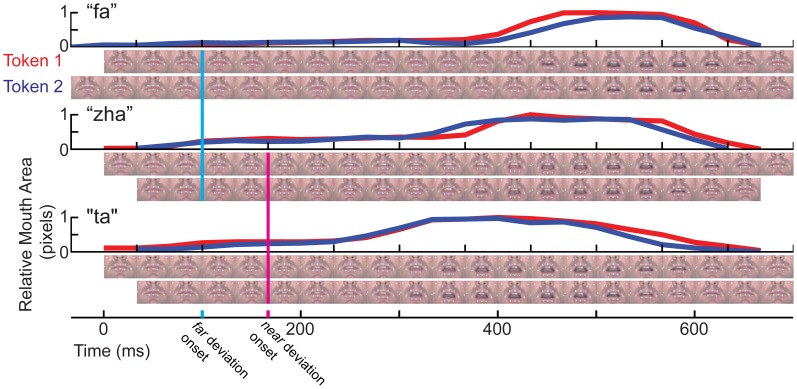
**Temporal kinematics of the syllables**. For each syllable, “fa,” “zha,” and “ta,” mouth opening area was measured in pixels, normalized to the range 0 (minimum for that syllable) to 1 (maximum for that syllable). Below each mouth opening graph are two rows of video images, one for each token of the stimulus. The images are cropped to show only the mouth area. The full face was shown to the participants in gray-scale. The vertical line in cyan marks the time of deviation for “zha” vs. “fa.” The magenta vertical line marks the time of deviation for “zha” vs. “ta.”

The two tokens of each consonant (e.g., “zha”) varied somewhat in their kinematics, so temporal alignments had to be defined prior to averaging the EEG data. We developed a method to align tokens of each syllable. Video clips were compared frame by frame separately for “zha,” “fa,” and “ta.” In addition, mouth opening area was measured as the number of pixels encompassed within a manual tracing of the vermillion border in each frame of each stimulus. Visual speech stimulus information is widely distributed on the talking face (Jiang et al., 2007a), but mouth opening area is a gross measure of speech stimulus kinematics. Figure 2 shows the mouth-opening area and video of the lips for the three different consonant-vowel (CV) stimuli and the two different tokens of each of them. The stimuli began with a closed neutral mouth and face, followed by the gesture into the consonant, followed by the gesture into the /a/ vowel (“ta,” “fa,” “zha”). Consonant identity information develops across time and continues to be present as the consonant transitions into the following vowel. The steep mouth opening gesture into the vowel partway through the stimulus was considered a possible landmark for temporal alignment, because it is a prominent landmark in the mouth area trace, but using this landmark in some cases brought the initial part of the consonant into gross misalignment. The frames comprising the initial gesture into the consonant were chosen to be the relevant landmark for alignment across tokens, because they are the earliest indication of the consonant identity (Jesse and Massaro, 2010).

The question was then, when did the image of one consonant (e.g., “fa”) deviate from the image of the other (e.g., “zha”). The MMN is typically elicited by stimulus deviation, rather than stimulus onset (Leitman et al., 2009), and this deviation onset point is used to characterize the relative timing of the vMMN. Typically, ERPs to visual stimuli require steep visual energy change (Besle et al., 2004), but visual speech stimulus onset can be relatively slow-moving, depending on the speech phonetic features. Careful examination of the videos shows that differences in the tongue are visible across the different consonants. The “zha” is articulated by holding the tongue in a quasi-steady-state somewhat flattened position in the mouth. This articulation is expected to take longer to register as a deviation, because of its subtle initial movement. The “ta” and “zha” stimuli vary primarily in terms of tongue position, which is visible but difficult to discern without attention to the tongue inside the mouth aperture. The deviation onset point here was defined as the first frame at which there was a visible difference across consonants. The 0-ms points in this report are set at the relevant deviation point and vMMN times are reported relative to the deviation onset.

### Procedures

#### Discrimination pre-test

To confirm the discriminability of the consonants comprising the critical contrasts in the EEG experiment, participants carried out a *same-different* perceptual discrimination task that used “zha”—“fa”, and “zha”—“ta” *different* stimulus pairs. The two tokens of each syllable were combined in each of four possible ways and in both possible orders. *Same* pairs used different tokens of the same syllable, so that accurate discrimination required attention to consonant category. This resulted in six unique *same* pairs and 16 unique *different* pairs. To reduce the difference in number of *same* pairs vs. the number of *different* pairs, the *same* pairs were repeated, resulting in 12 *same* pairs and 16 *different* pairs per block, for a total of 28 pairs per block. During each trial, the inter-stimulus interval was filled by a morph transition from the end of the first token to the start of the second lasting 267 ms. Instructions emphasized that the tokens might differ in various ways, but that the task was to determine if the initial consonants were the same or different. Eleven blocks of pseudo-randomly ordered trials were presented. The first block was used for practice to ensure the participants' familiarity with the task, and it was not analyzed.

#### vMMN procedure

EEG recordings were obtained during an oddball paradigm in which standard, deviant, and target stimuli were presented. If one stimulus category is used as the standard and a different category stimulus is used as the deviant in deriving the vMMN, the vMMN also contains a response to the physical stimuli (Czigler et al., 2002). In order to compare ERPs to standards vs. deviants, holding the stimulus constant, each stimulus was tested in the roles of deviant and standard across different recording blocks (Table 1)^1^.

**Table 1:** **Syllables included in each of four block types**.

**Block type**	**Standard**	**Deviant**	**Target**	**Dissimilarity^a^**
1	“zha”	“ta”	“va”	2.28
2	“ta”	”zha”	“va”	2.28
3	“zha”	“fa”	“ha”	4.04
4	“fa”	“zha”	“ha”	4.04

Each block had a standard syllable, a deviant syllable and a target syllable.

a Dissimilarity measures the difference between the standard and the deviant syllable.

EEG recording comprised 40 stimulus blocks divided across four block types (Table 1). Each block type had one *standard* consonant (i.e., “zha,” “fa,” or “ta”), one *deviant* consonant (i.e., “zha,” “fa,” or “ta”), and one *target* consonant (i.e., “ha,” or “va”). The “zha” served as *deviant* or *standard* with either “fa” or “ta.” Thus, four vMMNs were sought: (1) “zha” in the context of “ta” (*near*); (2) “ta” in the context of “zha” (*near*); (3) “zha” in the context of “fa” (*far*); and (4) “fa” in the context of “zha” (*far*). Each vMMN was based on 10 stimulus blocks with the vMMN stimulus in either deviant or standard role. During each block, a *deviant* was always preceded by five to nine *standards*. At the beginning of a block, the *standard* was presented 9 times before the first *deviant*. The inter-stimulus-interval was measured as the duration of the morphs between the end of a stimulus and the beginning of the next, which was 267 ms.

To ensure that the visual stimuli were attended, participants were instructed to monitor the stimuli carefully for a *target* syllable. At the start of each block, the target syllable was identified by presenting it six times in succession. A *target* was always preceded by three to five *standards*. Participants were instructed to press a button upon detecting the target, which they were told would happen rarely. In each block, the *target* was presented four times, and the *deviant* was presented 20 times. In all, 85.4% of stimuli in a block were standards, 12.1% were deviants and 2.4% were targets. This corresponded to 200 *deviant* trials and ~1400 *standard* trials per contrast per subject. The first *standard* trial following either a *deviant* trial or a *target* trial was discarded from analysis, because a standard following something other than a standard might generate a MMN (Sams et al., 1984; Nousak et al., 1996). This resulted in 1160 *standard* trials for computing the vMMN.

Participants were instructed to take self-paced breaks between blocks, and longer breaks were enforced every 10 blocks. Recording time was ~4.5 h per participant. After EEG recording, electrode locations recorded were for each subject using a 3-dimensional digitizer (Polhemus, Colchester, Vermont).

### EEG recording and offline data processing

EEG data were recorded using a 62-electrode cap that was configured with a modified 10–20 system for electrode placement. Two additional electrodes were affixed at mastoid locations, and bipolar EOG electrodes were affixed above and below the left eye and at the external canthi of the eyes to monitor eye movements. The EEG was amplified using a high input impedance amplifier (SynAmps 2, Neuroscan, NC). It was digitized at 1000 Hz with a 200 Hz low-pass filter. Electrode impedances were measured, and the inclusion criterion was 35 kOhm.

Offline, data were band-pass filtered from 0.5 to 50 Hz with a 12-dB/octave rolloff FIR zero phase-shift filter using EDIT 4.5 software (Neuroscan, NC). Eyeblink artifacts were removed using EDIT's blink noise reduction algorithm (Semlitsch et al., 1986). Data were epoched from 100 ms before video onset to 1000 ms after video onset. Epochs were baseline-corrected by subtracting the average of the voltage measurements from −100 to +100 ms for each electrode and then average-referenced.

Artifact rejection and interpolation were performed using custom scripts calling functions in EEGLAB (Delorme and Makeig, 2004). Epochs in which no electrode voltage exceeded 50 μV at any point in the epoch were included. For those epochs in which only one electrode exceeded the 50 μV criterion, the data for that electrode were interpolated using spherical spline interpolation (Picton et al., 2000). This procedure resulted in inclusion of 91% of the EEG sweeps. To correct for variation in electrode placement between subjects, individual subject data were projected onto a group average set of electrode positions using spherical spline interpolation (Picton et al., 2000).

### Analyses of discrimination data

*Same-different* discrimination sensitivity was measured with *d*′ (Green and Swets, 1966). The hit rate was the proportion *different* responses to trials with different syllables. The false alarm rate was the proportion *different* responses for same pairs. If the rate was zero it was replaced with 1/(2*N*), and if it was one it was replaced by 1–1/(2*N*), where *N* is the number of trials (Macmillan and Creelman, 1991). Because this is a *same-different* design, *z*(*hit rate*)—*z*(*false alarm rate*) was multiplied by $\sqrt{2}$ (Macmillan and Creelman, 1991).

Target detection during the EEG task was also evaluated using *d*′, but the measure was *z*(*hit rate*)—*z*(*false alarm rate*). A response within 4 s of the target presentation was considered a *hit*, and a *false alarm* was any response outside this window. All non-target syllables were considered distracters for the purpose of calculating a false alarm rate. To assess differences in target detection across blocks, *d*′ was submitted to repeated-measures ANOVA.

### Analyses of EEG data

#### Overview

*A priori*, the main hypothesis was that visual speech stimuli are processed by the visual system to the level of representing the exogenous visual syllables. Previous research had suggested that there was specialization for visual speech stimuli by left posterior temporal cortex (Campbell et al., 2001; Bernstein et al., 2011; Campbell, 2011; Nath and Beauchamp, 2012). Previous research also suggested that there was specialization for non-speech face motion by right posterior temporal cortex (Puce et al., 1998, 2000, 2007; Bernstein et al., 2011). Therefore, the *a priori* anatomical regions of interest (ROI) were the bilateral posterior temporal cortices. However, rather than merely selecting electrodes of interest (EOI) over scalp locations approximately over those cortices and carrying out all analyses with those EOIs, a more conservative, step-by-step approach was taken, which allowed for the possibility that deviation detection was carried out elsewhere in cortex (e.g., Sams et al., 1991; Möttönen et al., 2002).

In order first to test for reliable stimulus deviation effects, independent of temporal window or spatial location, global field power (GFP; Lehmann and Skrandies, 1980; Skrandies, 1990) measures were compared statistically across standard vs. deviant for each of the four different vMMN contrasts. The GFP analyses show the presence and temporal interval of a deviation response anywhere over the scalp. The first 500 ms post-stimulus deviation was examined, because that interval was expected to encompass any possible vMMN.

Next, source analyses were carried out to probe whether there was evidence for stimulus processing by posterior temporal cortices, consistent with previous fMRI results on visual speech perception (Bernstein et al., 2011). Distributed dipole sources (Tadel et al., 2011) were computed for the responses to standard stimuli and for the vMMN waveforms. These were inspected and compared with the previous Bernstein-et-al. results and also with results from previous EEG studies that presented source analyses (Bernstein et al., 2008; Ponton et al., 2009). The inspection focused on the first 500 ms of the source models.

After examining the source models, EOIs were sought for statistical testing of vMMNs, taking into account the ERPs at individual electrode locations. For this level of analysis, an approach was needed to guard against double-dipping, that is, use of the same results to select and test data for hypothesis testing (Kriegeskorte et al., 2009). Because we did not have an independent localizer (i.e., an entirely different data set with which to select EOIs), as is recommended for fMRI experiments, we ran analyses on several different electrode clusters over posterior temporal cortices. Because all those results were highly similar, only one set of EOI analyses are presented here.

A coincident frontal positivity has also been reported for Fz and/or Cz in conjunction with evidence for a vMMN (Czigler et al., 2002, 2004). The statistical tests for the vMMN were carried out separately on ERPs from electrodes Fz and Cz to assess the presence of a frontal MMN. These tests also served as a check on the validity of the EOI selection. Fz and Cz electrodes are commonly used for testing the auditory MMN (Näätänen et al., 2007). If the same results were obtained on Fz and Cz as with the EOIs, the implication would be that EOI selection was biased toward our hypothesis that the posterior temporal cortices are responsible for visual speech form representations. The results for Fz and Cz were similar to each other but different from the EOI results, and only the Fz results are presented here. None of the Cz results were statistically reliable. ERPs evoked by target stimuli were not analyzed, because so few target stimuli were presented.

#### Global field power

GFP (Lehmann and Skrandies, 1980; Skrandies, 1990) is the root mean squared average-referenced potential over all electrodes at a time sample. The GFP was calculated for each standard and deviant ERP per stimulus and per subject. The analysis window was 0–500 ms post stimulus deviation. Statistical analysis of group mean GFP differences between standard and deviant, within syllable, used randomization testing (Blair and Karniski, 1993; Nichols and Holmes, 2002; Edgington and Onghena, 2007) of the null hypothesis of no difference between the evoked response when the stimulus was a *standard* vs. the evoked response when the stimulus was a *deviant*. The level of re-sampling was the individual trial.

Surrogate mean GFP measures were generated for each subject by permuting the single-trial labels (i.e., *standard* or *deviant*) 1999 times and then computing mean GFP differences (*deviant* minus *standard*) for these permutation samples. These single-subject permutation mean GFP differences were averaged across subjects to obtain a permutation distribution of group mean GFP differences within the ERPs for a particular syllable. To avoid bias due to using a randomly generated subset of the full permutation distribution, the obtained group mean GFP difference was included in the permutation distribution, resulting in a total of 2000 entries in the permutation distribution. The *p*-value for a given time point was calculated as the proportion of surrogate group mean GFP difference values in the permutation distribution that were as or more extreme than the obtained group mean GFP difference, resulting in a two-tailed test.

To correct for multiple comparisons over time, a threshold length of consecutive *p*-values <0.05 was established (Blair and Karniski, 1993; Groppe et al., 2011). The threshold number of consecutive *p*-values was determined from the permutation distribution generated in the corresponding uncorrected test. For each entry in the permutation distribution, a surrogate *p*-value series was computed as though that entry were the actual data. Then, the largest number of consecutive *p*-values <0.05 in that surrogate *p*-value series was computed for each permutation entry. The threshold number of consecutive *p*-values was the 95th percentile of this null distribution of run lengths. This correction, which offers weak control over family-wise error rate and is appropriate when effects persist over many consecutive samples (Groppe et al., 2011), is similar to one used with parametric statistics (Guthrie and Buchwald, 1991) but requires no assumptions or knowledge about the autocorrelation structure of the underlying signal or noise.

#### EEG distributed dipole source models

EEG sources were modeled with distributed dipole source imaging using Brainstorm software (Tadel et al., 2011). In lieu of having individual anatomical MRI data for source space and forward modeling, the MNI/Colin 27 brain was used. A boundary element model (Gramfort et al., 2010) was fit to the anatomical model using a scalp model with 1082 vertices, a skull model with 642 vertices, and a brain model with 642 vertices. The cortical surface was used as the source space, and source orientations were constrained to be normal to the cortical surface. Cortical activity was estimated using depth-weighted minimum-norm estimation (wMNE; Baillet et al., 2001).

EEG source localization is generally less precise than some other neuroimaging techniques (Michel et al., 2004). Simulations comparing source localization techniques resulted in a mean localization error of 19.6 mm when using a generic brain model (Darvas et al., 2006), as was done here. Similar methods were used here, so the estimate of localization errors is ~20 mm. Therefore, the source solutions found here serve as useful visualization tools and for EOI selection but are not intended for making conclusion related to precise anatomical localization.

#### vMMN analyses

The vMMN analyses used the same general approach as the approach to the GFP analyses rather than the more pervasive analysis of difference waveforms. To assess the reliability of the vMMNs for each stimulus, the average of the ERP for the EOIs for the token-as-standard was compared with the average of the ERPs for the token-as-deviant using a standard paired-samples permutation test (Edgington and Onghena, 2007) with the subject mean ERP as the unit of re-sampling. A threshold number of consecutive *p*-values <0.05 was established to correct for multiple comparisons using the same criterion (Blair and Karniski, 1993) as described above for the GFP analyses. The EOI cluster results that are presented are from the clusters left P5, P3, P1, PO7, PO5, and PO3, and right P2, P4, P6, PO4, PO6, and PO8^2^. We also carried out comparisons of the difference waveforms across *near* vs. *far* contrasts. These were a general check on the hypothesis that *far* contrasts were different from *near* contrasts.

In some cases in which a vMMN is observed, a coincident frontal positivity has also been reported for Fz and/or Cz (Czigler et al., 2002, 2004). The statistical tests for the vMMN were carried out separately on ERPs from electrodes Fz and Cz to assess the presence of a frontal MMN.

## Results

### Behavioral results

The purpose of testing behavioral discrimination was to assure that the stimulus pair discriminability was predicted correctly. The 49 screened participants were tested, and the EEG data from 11 of them are reported here. Discrimination *d*′ scores were compared across groups (included vs. excluded participants) using analysis of variance (ANOVA) with the within-subjects factor of stimulus distance (*near* vs. *far*) and between-subjects factor of group (included vs. excluded). The groups were not reliably different, and group did not interact with stimulus distance.

*Far* pairs were discriminated better than *near* pairs, *F*_(1, 47)_ = 591.7, *p* < 0.001, mean difference in *d*' = 3.13. Within the EEG group, mean *d*′ for the *far* stimulus pairs was reliably higher than for the *near* stimulus pairs, paired-*t*_(10)_ = 12.25, *p* < 0.001, mean difference in *d*' = 3.02. Mean *d*′ was reliably above chance for both *near, t*_(10)_ = 8.09, *p* < 0.001, *M* = 1.40, and *far, t*_(10)_ = 15.62, *p* < 0.001, *M* = 4.51, stimulus pairs.

Detection *d*′ of “ha” or “va” during EEG recording was high, group mean *d*' = 4.83, range [3.83, 5.91]. The two targets were detected at similar levels, *paired-t*_(10)_ = 0.23, *p* = 0.82. For neither target syllable was there any effect of which syllable was the standard in the EEG recording block.

#### ERPs across vMMN stimulus pairs

The ERP group mean data sets for the four stimulus pairs were inspected for data quality. Figures S1–S2 show the montages for each of the vMMN data sets.

#### GFP results

**Figure 3:**
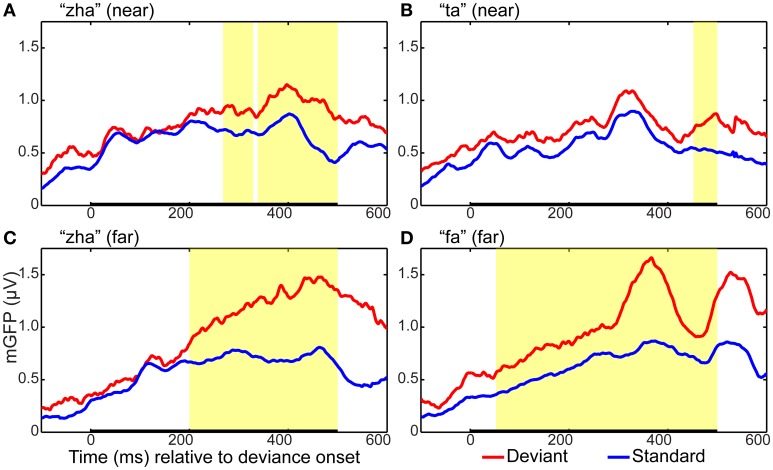
**Global field power plots for the four vMMN contrasts**. Group mean global field power (mGFP) evoked by the standard and the deviant are shown for **(A)** “zha” in the *near* context, **(B)** “ta” in the *near* context, **(C)** “zha” in the *far* context, and **(D)** “fa” in the *far* context. The time axes show time relative to the onset of stimulus deviation. Highlighted regions show times of statistically significant difference (*p* < 0.05) between standard and deviant GFPs, as determined by a permutation test corrected for multiple comparisons over time. Statistical comparisons were performed over the times indicated by the heavy black line along the time axis. This time window was selected to include the expected time for a vMMN evoked by the consonant part of the syllable.

GFP measures were computed for each standard and deviant syllable. Holding syllable constant, the standard vs. deviant GFP was compared to determine whether and, if so, when a reliable effect of stimulus deviance was present in each of the four stimulus conditions (i.e., “zha” in the *near* context, “zha” in the *far* context, “fa” a *far* contrast, and “ta” a *near* contrast). All of the stimulus contrasts resulted in reliable effects. Figure 3 summarizes the GFP results for each vMMN. The reliable GFP difference for “zha” in the *far* context was 200–500 ms post-deviation onset. For “zha” in the *near* context, there were two intervals of reliable difference, 268–329 and 338–500 ms post-deviation onset. The reliable difference for “fa” was 52–500 ms post-deviation onset. The reliable difference for “ta” was 452–500 ms post-deviation onset.

#### Distributed dipole source models

**Figure 4:**
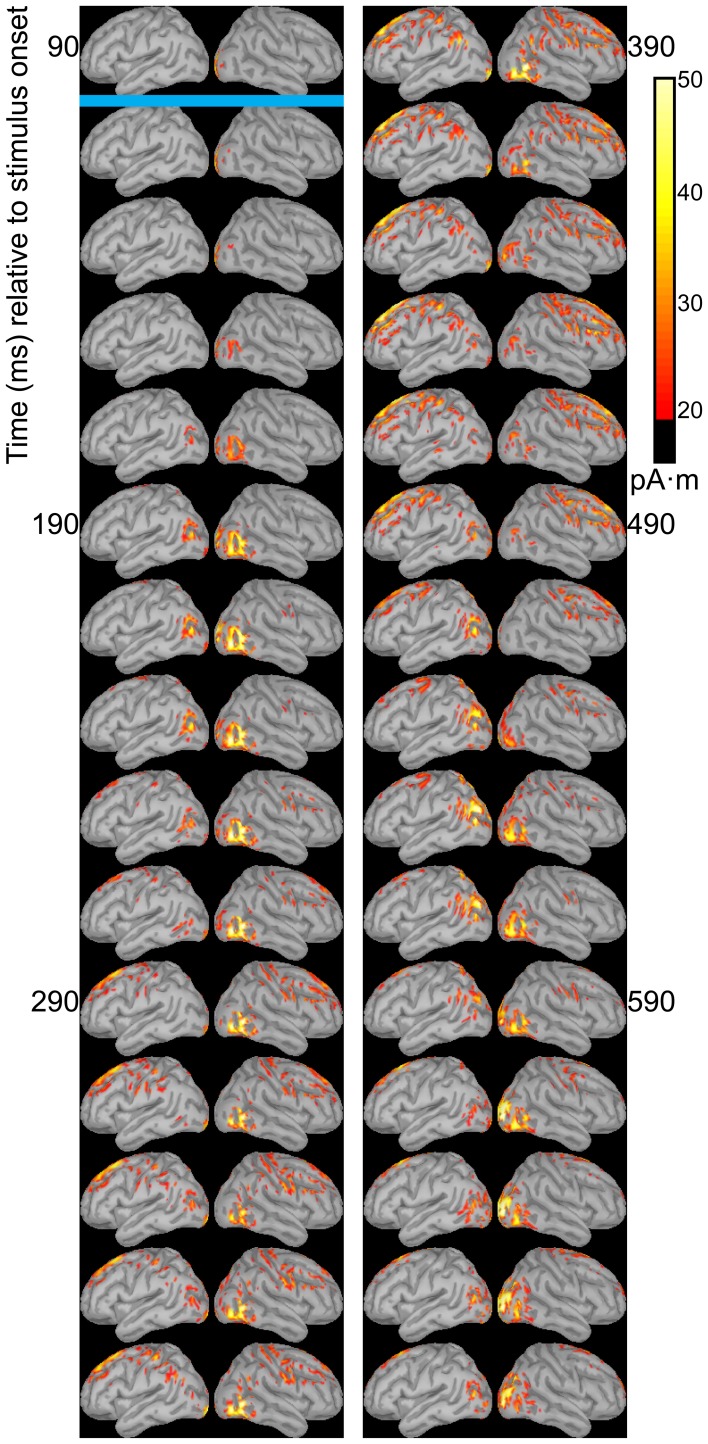
**Source images for “fa” standard**. Images show the depth-weighted minimum norm estimate of dipole source strength constrained to the surface of the cortex using a boundary element forward model and a generic anatomical model at 20-ms intervals starting from 90 ms after onset of visible motion for the group mean ERPs for syllable “fa” as *standard*. The time indicated by the cyan bar indicates the time at which “fa” visibly differs from “zha.” Images are thresholded at 20 pA·m. Initial activity is in the occipital cortex. At 150 ms after syllable onset, the bilateral posterior temporal activity begins that lasts until 290 ms in the left hemisphere and until 490 ms in the right hemisphere. Activation in the right posterior temporal cortex is more widespread and inferior to that on the left. Fronto-central activity is visible from 250 to 510 ms post-stimulus onset.

**Figure 5:**
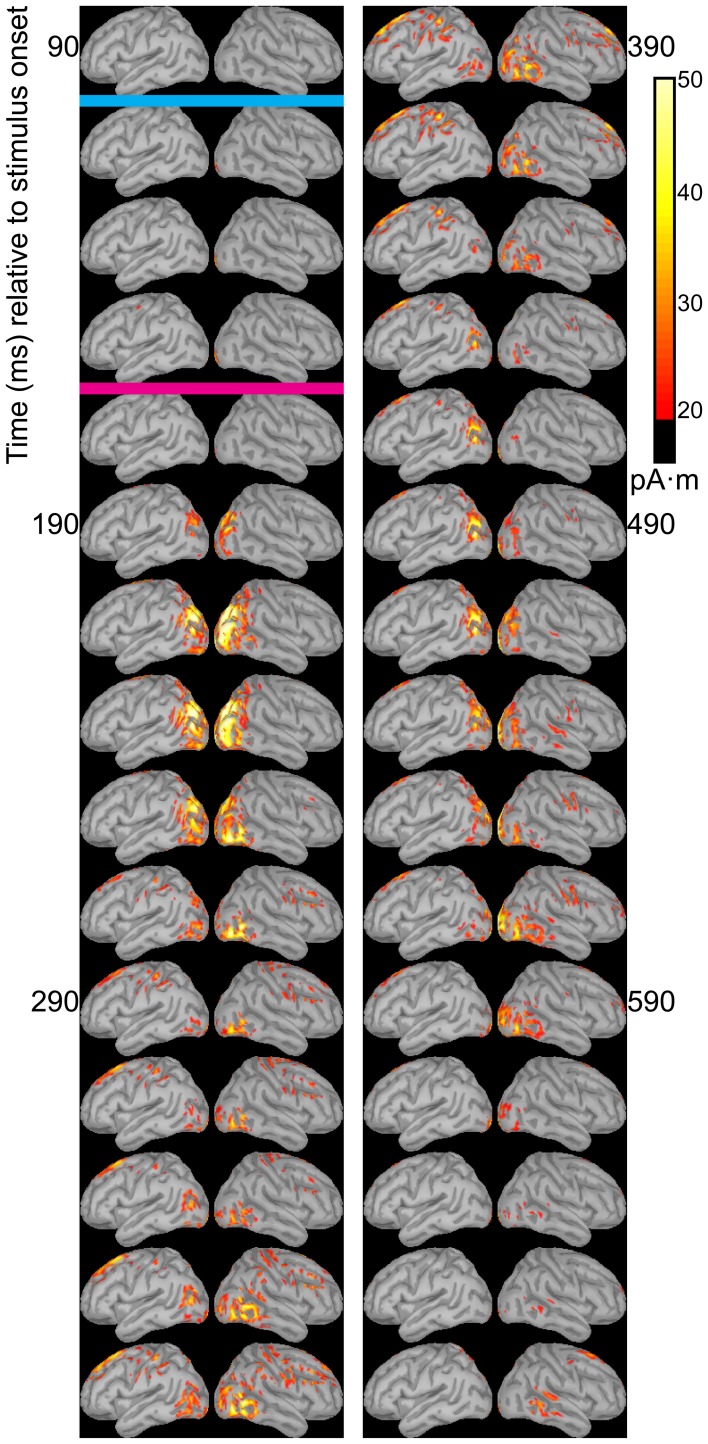
**Source images for “zha” standard**. Images show the depth-weighted minimum norm estimate of dipole source strength constrained to the surface of the cortex using a boundary element forward model and a generic anatomical model at 20-ms intervals starting from 90 ms after the onset of visible motion for the group mean ERPs for syllable “zha” as *standard*. The cyan bar indicates the time at which “zha” visibly differs from “fa,” and the magenta bar indicates the time at which “zha” visibly differs from “ta.” Images are thresholded at 20 pA·m. Initial activity is in the occipital cortex. At 190 ms after syllable onset, strong, widespread bilateral posterior temporal activity begins that lasts until 290 ms, with weaker activations recurring through 610 ms post-stimulus onset. Activation in the right posterior temporal cortex is more widespread and inferior to that on the left. Fronto-central activity is visible from 270 to 490 ms post-stimulus onset.

**Figure 6:**
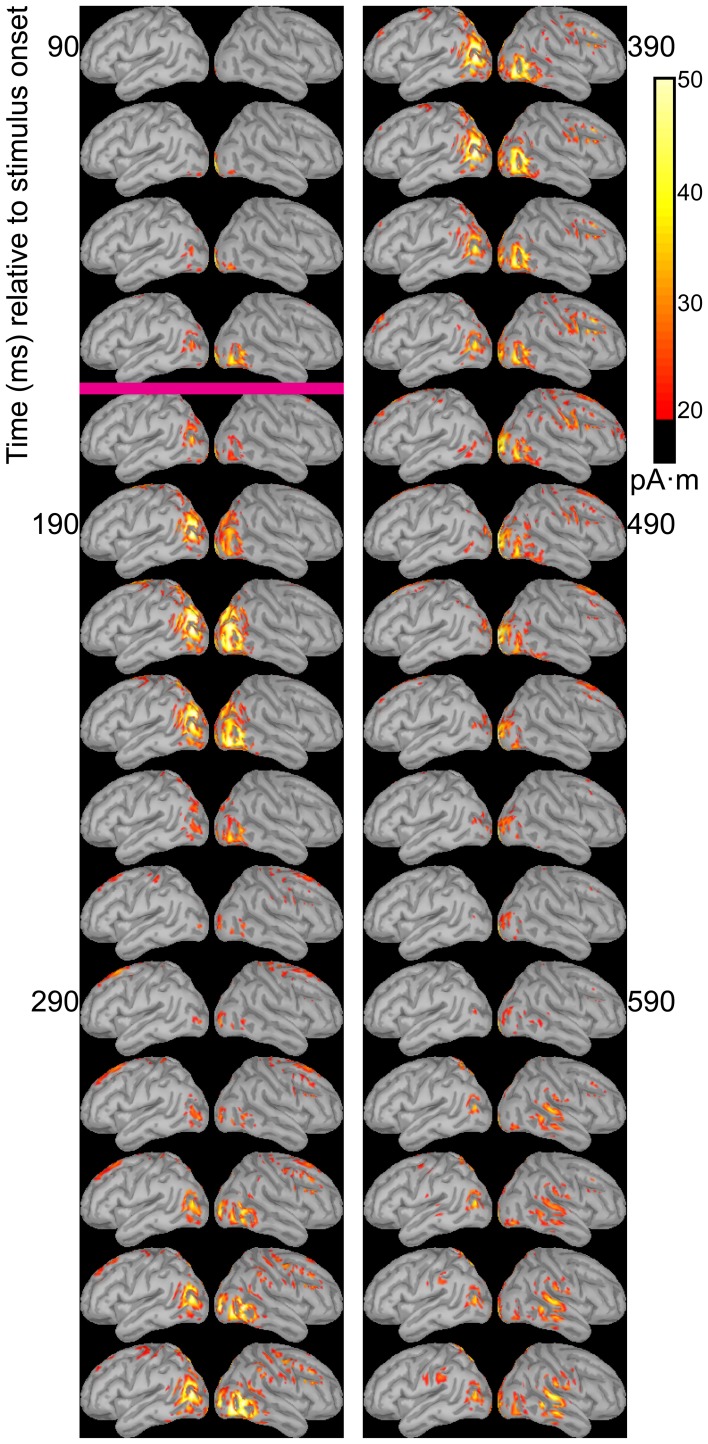
**Source images for “ta” standard**. Images show the depth-weighted minimum norm estimate of dipole source strength constrained to the surface of the cortex using a boundary element forward model and a generic anatomical model at 20-ms intervals starting from 90 ms after the onset of visible motion for the group mean ERPs for syllable “ta” as *standard*. The magenta bar indicates the time at which “ta” visibly differs from “zha.” Images are thresholded at 20 pA·m. Initial activity is in occipital cortex. At 130 ms after syllable onset, bilateral posterior temporal activity begins that fades by 250 ms post-stimulus onset, but then recurs from 330 to 590 ms on the right and from 330 to 470 ms on the left. Fronto-central activity is visible from 270 to 470 ms post-stimulus onset.

Dipole source models were computed using ERPs obtained with standard stimuli (“zha,” “fa,” and “ta”) in order to visualize the spatiotemporal patterns of exogenously driven responses to the stimuli. Figures 4–6 show the dipole source strength at 20-ms intervals starting from 90 ms after onset of visible motion until 670 ms for the group mean ERPs. The images are thresholded to only show dipole sources stronger than 20 pA·m. The figures show images starting at 90 ms post-stimulus onset, because no suprathreshold sources were obtained earlier. The images continue through 690 ms to indicate that posterior activity rises and falls within the interval, as would be expected in response to a temporally unfolding stimulus.

The right hemisphere overall appeared to have stronger and more sustained responses focused on posterior temporal cortex. Additionally, the right posterior temporal activation was more widespread but with a more inferior focus compared to that in left posterior temporal cortex. Variations in the anatomical locations of the foci of activity across Figures 4–6 suggest that the possibility that activation sites varied as a function of syllable. But these cannot be interpreted with confidence given the relatively low level of spatial resolution of these distributed dipole source models.

The temporal differences across syllable are more interpretable. Variation across syllables is attributed to differences in stimulus kinematics. The “fa” standard (Figure 4) resulted in sustained right hemisphere posterior temporal activity from ~120 to 490 ms relative to stimulus onset and sustained left hemisphere posterior temporal activity from ~170 to 270 ms. The “zha” standard (Figure 5) resulted in sustained right hemisphere posterior temporal activity from ~190 to 430 ms and sustained left hemisphere posterior temporal activity from ~190 to 390 ms. The “ta” standard (Figure 6) resulted in sustained right hemisphere posterior temporal activity from ~150 to 250 ms and sustained left hemisphere posterior temporal activity from ~150 to 230 ms. The shorter period of sustained activity for “ta” vs. “fa” and “zha” can be explained by its shorter (fewer frames) initial articulatory gesture (Figure 2).

Some fronto-central and central activity emerged starting 220 to 280 ms post-stimulus onset, particularly with “zha” and “fa.” No other prominent activations were obtained elsewhere during the initial periods of sustained posterior temporal activity.

Dipole source models were also computed on the vMMN difference waveforms (Figures S3–S6), resulting in lower signal strength in posterior temporal cortices in comparison with models based on the standard ERPs. The models support the presence of deviance responses in those cortical areas and higher right posterior activity for *far* contrasts than *near* contrasts. All of the difference waveform models demonstrate patterns of asymmetric frontal activity with greatest strength generally beyond 200 ms post-deviation that seems attributable to attention to the deviant.

#### vMMN results

ERPs of EOI clusters for each syllable contrast and hemisphere were submitted to analyses to determine the reliability of the deviance responses. Thus, there were four vMMN analyses per hemisphere. They were for “zha” in its *near* or *far* context, “fa” in the *far* context, and “ta” in the *near* context. Summaries of the results are given in Table 2. The duration (begin points to end points) of reliable deviance responses varied across syllables (from 50 to 185 ms) and varied in mean voltage (from −0.35 to −0.85 μV).

**Table 2:** **Summary of reliable vMMNs**.

**Syllable (contrast)**	**Electrode(s)**	**Begin (ms)**	**End (ms)**	**Duration (ms)**	***p*-value^a^**	**Mean (μV)^b^**
Zha (Far)	LPT	322	497	176	0.010	−0.67
	RPT	324	500	177	0.006	−0.85
Zha (Near)	RPT	239	288	50	0.049	−0.35
Fa (Far)	LPT	251	435	185	0.006	−0.54
	RPT	300	442	143	0.002	−0.83
Ta (Near)	RPT	449	500	52	0.041	−0.52

All times are relative to deviance onset. LPT, left posterior temporal; RPT, right posterior temporal.

a The p-value corresponds to the entire indicated time window and is corrected for multiple comparisons over time.

b The mean is the group average deviant minus standard, averaged over the period from the begin to end points.

**Figure 7:**
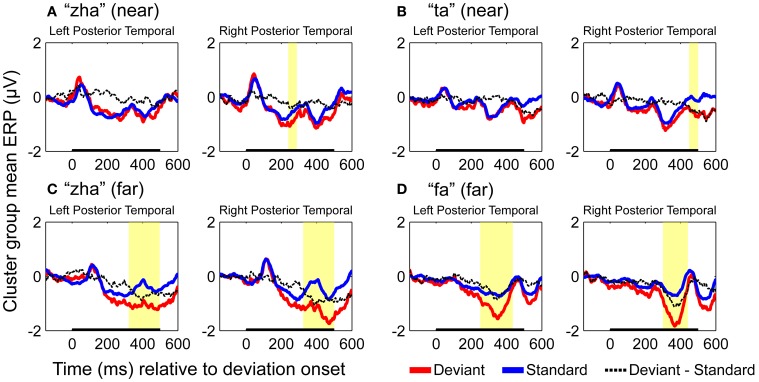
**Group mean ERPs and vMMN analyses for posterior temporal EOI clusters. (A)** VMMN results for “zha” (near) over left and right posterior temporal cortices. **(B)** VMMN results for “ta” near over left and right posterior temporal cortices. **(C)** VMMN results for “zha” (far) over left and right posterior temporal cortices. **(D)** VMMN results for “fa” (far) over left and right posterior temporal cortices. Time shown is relative to stimulus deviation onset. Statistical comparisons were performed over the times indicated by the heavy black line along the time axis. Highlighted regions denote statistically significant (*p* < 0.05) differences of ERPs evoked by the stimulus as deviant vs. standard, corrected for multiple comparisons over time. Reliable differences were obtained for the right EOI means with all four syllable contrasts. Reliable differences were obtained for the left EOI means with only two *far* vMMN contrasts.

Figure 7 shows the statistical results for the EOI cluster waveforms for each contrast and hemisphere. The theoretically predicted results were obtained. All of the right-hemisphere contrasts resulted in reliable deviance responses. They were “zha” in the *near* context from 239 to 288 ms post-deviation onset, “zha” in the *far* context from 324 to 500 ms post-deviation onset, “ta” from 449 to 500 ms post-deviation onset, and “fa” from 300 to 442 ms post-deviation onset. Only the *far* contrasts resulted in reliable left-hemisphere deviance responses. They were “zha” in the *far* context from 322 to 497 ms post-deviation onset and “fa” from 251 to 435 ms post-deviation onset.

#### Comparison of far vs. near vMMNs

Difference waveforms were computed using the standard type of approach to the vMMN, that is, by subtracting the EOI cluster ERPs to standards from the response to deviants for each stimulus contrast and hemisphere on a per-subject basis. The magnitudes of the vMMN waveforms were then compared between *far* and *near* contrasts using the resampling method that was applied to the analyses of standards vs. deviants.

**Figure 8:**
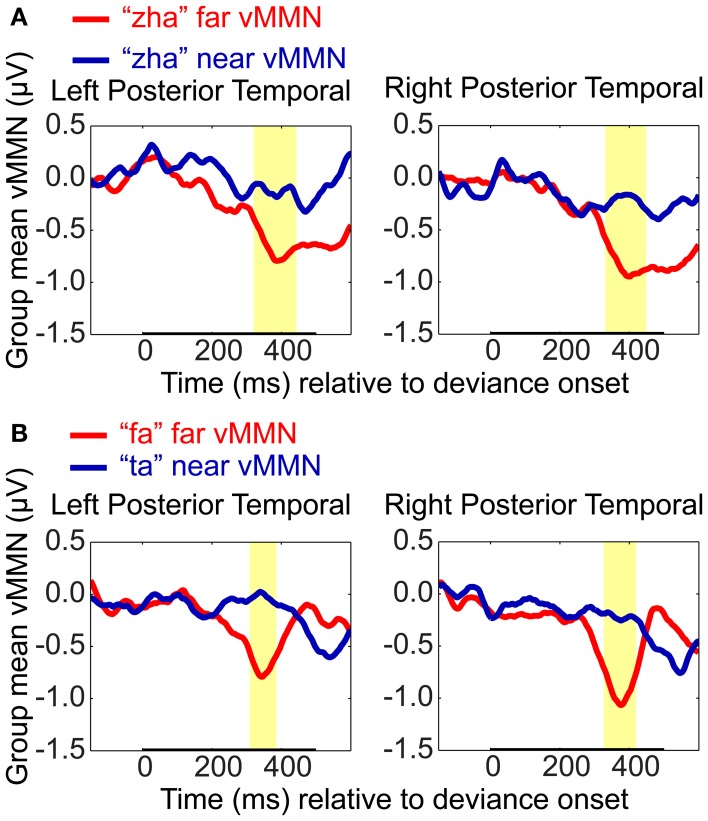
**VMMN comparisons**. Group mean vMMN difference waves (deviant minus standard) from EOI means were compared to test whether syllable distance (*far* vs. *near*) predicted relative vMMN magnitude. Comparisons were **(A)** “zha” in the *far* context vs. “zha” in the *near* context, and **(B)** “fa” (*far* context) vs. “ta” (*near* context). Statistical comparisons were performed over the times indicated by the heavy black line along the time axis. Highlighted regions denote statistically significant (*p* < 0.05) differences in the vMMNs corrected for multiple comparisons over time. For display purposes only, difference waves were smoothed with a 41-sample moving average.

**Figure 9:**
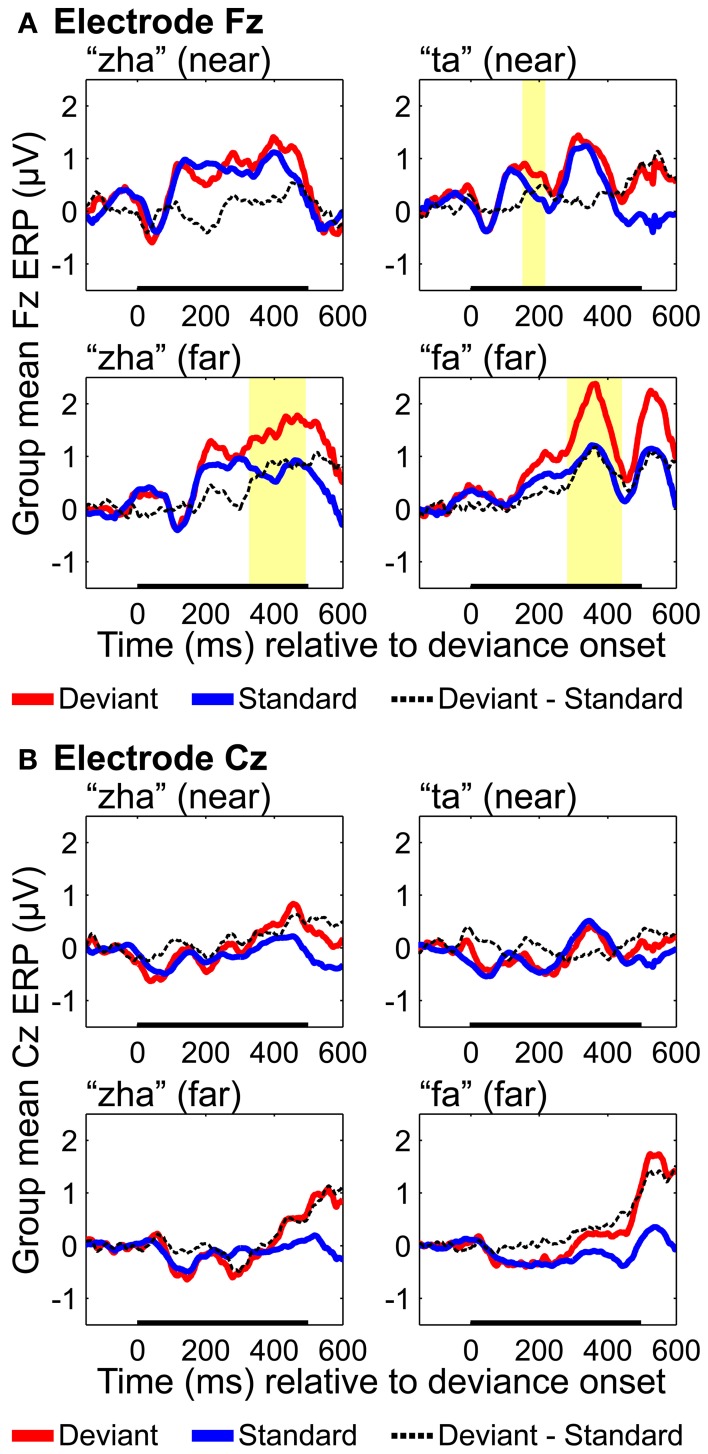
**Group mean ERPs and MMN analyses for (A) electrode Fz and (B) electrode Cz**. Group mean ERPs are shown for “zha” in the *near* context, “ta” in the *near* context, “zha” in the *far* context, and “fa” in the *far* context. Times shown are relative to stimulus deviation onset. Highlighted time regions show statistically significant (*p* < 0.05) differences of ERP evoked by the deviant from the ERP evoked by the standard, corrected for multiple comparisons over time. Statistical comparisons were performed for the times indicated by the heavy black line along the time axis. Reliable positive differences (deviant vs. standard) were obtained on electrode Fz for the two *far* syllable contrasts and for the *near* syllable contrast “ta.” No reliable differences were obtained on electrode Cz.

The “zha” *near* and *far* vMMN waveforms were found to be reliably different (Figure 8). On the left, the difference wave for “zha” in the *far* context was reliably larger (i.e., more negative) than for “zha” in the *near* context (320 to 443 ms post-deviation onset), not unexpectedly as the *near* context did not result in an observable vMMN. On the right, the difference wave was also reliably larger for “zha” in the *far* context (from 331 to 449 ms post-deviation onset), although both contexts were effective. The results were similar when the vMMN waveforms were compared between “fa” vs. “ta” (Figure 9). On the left, the difference wave for “fa” was reliably larger than for “ta” (309–386 ms post-deviation onset). On the right, the difference wave was also reliably larger for “fa” (from 327 to 420 ms post-deviation onset).

#### Fronto-central results

ERPs were analyzed based on recordings from electrodes Fz and Cz, because these electrodes are typically used to obtain an auditory MMN (Kujala et al., 2007), but positivities on these electrodes have been reported for vMMNs (Czigler et al., 2002, 2004). Results with Fz (Figure 9) showed reliable effects for “ta,” “fa,” and “zha” *far*. None of the Cz results were reliable (Figure 9). Reliable differences with the deviant ERPS more positive were found on Fz for both of the *far* contrasts, from 282 to 442 ms post-deviation onset for “fa” and from 327 to 492 ms post-deviation onset for “zha” in the *far* context. These positive differences occur at similar times and with opposite polarity as the posterior temporal vMMNs. A reliable positivity was also obtained for “ta” from 151 to 218 ms post-deviation onset, but no reliable difference was obtained for “zha” in the *near* context.

## General discussion

This study investigated the brain's response to visual speech deviance, taking into account that (1) responses to stimulus deviants are considered to be generated by the cortex that represents the stimulus (Winkler and Czigler, 2012), and (2) that there is evidence that exogenous visual speech processing is lateralized to left posterior temporal cortex (Campbell, 1986; Campbell et al., 2001; Bernstein et al., 2011). Taken together these observations imply that the right and left posterior temporal cortices represent visual speech stimuli differently, and therefore that their responses to stimulus deviance should differ.

We hypothesized that the right posterior temporal cortex, for which there are indications of representing simple non-speech face gestures (Puce et al., 2000, 2003), would generate the deviance response to both perceptually *near* and perceptually *far* speech stimulus changes (Figure 1). In contrast, the left hemisphere, for which there are indications of specialization (Campbell et al., 2001; Bernstein et al., 2011; Campbell, 2011; Nath and Beauchamp, 2012) for representing the exogenous stimulus forms of speech, would generate the deviance response only to perceptually *far* speech stimulus changes. That is, it would be tuned to stimulus differences that are readily perceived as different consonants (Figure 1).

Two vMMNs were sought for *far* stimulus deviations (one for “zha” and one for “fa”), and two vMMNs were sought for *near* stimulus deviations (one for “zha” and one for “ta”). The “zha” stimulus was used to obtain a perceptually *near* and a perceptually *far* contrast in order to hold consonant constant across perceptual distances. Reliable vMMN contrasts supported the predicted hemispheric effects. The left-hemisphere vMMNs were obtained only with the highly discriminable (*far*) stimuli, but the right-hemisphere vMMNs were obtained with both the *near* and *far* stimulus contrasts. There were also reliable differences between vMMN difference waveforms as a function of perceptual distance, with larger vMMN difference waveforms associated with larger perceptual distances.

### Evidence for the vMMN deviance response with speech stimuli

Previous reports have been mixed concerning support for a posterior vMMN specific to visual speech form-based deviation (i.e., deviation based on the phonetic stimulus forms). An early study failed to observe any vMMN in a paradigm in which a single visual speech token was presented as a deviant and a single different speech token was presented as a standard (Colin et al., 2002). A more recent study (Saint-Amour et al., 2007) likewise failed to obtain a vMMN response.

In Colin et al. (2004), a posterior difference between the ERP evoked by a standard syllable and the ERP evoked by a deviant syllable was obtained on Oz (from 155 to 414 ms), but this difference was attributed to low-level (non-speech) stimulus differences and not to speech syllable differences, because the effect involved two different stimuli. A subsequent experiment controlling for stimulus difference found no vMMN for visual speech alone. For example, the original deviance detection could have arisen at a lower-level such as the temporal or spatial frequency differences between the stimuli, or it could have been the result of shifts in the talker's eye gaze across stimuli. A study by Möttönen et al. (2002) used magnetoencephalography (MEG) to record the deviance response with a single standard (“ipi”) vs. a single deviant (“iti”). The mismatch response was at 245–410 ms on the left and 245–405 ms on the right. But again, these responses cannot be attributed exclusively to deviance. They could be attributable to consonant change.

Winkler et al. (2009) compared the ERPs to a “ka” stimulus in its roles as standard vs. deviant and reported a late occipital difference response, and possibly also an earlier negative difference peak at 260 ms on occipital electrodes that did not reach significance. In their study, the vMMN is not attributable to lower-level stimulus attributes that changed.

Ponton et al. (2009) used a similar approach in attempting to obtain vMMNs for “ga” and “ba.” A reliable vMMN was obtained for “ba” only. The authors speculated that the structure of the “ga” stimulus might have impeded being able to obtain a reliable vMMN with it. The stimulus contained three early rapid discontinuities in the visible movement of the jaw, which might have each generated their own C1, P1, and N1 responses, resulting in the oscillatory appearance of the obtained vMMN difference waveforms. Using current density reconstruction modeling (Fuchs et al., 1999), the “ba” vMMN was reliably localized only to the right posterior superior temporal gyrus, peaking around 215 ms following stimulus onset. The present study suggests that the greater reliability for localizing the right posterior response could be due to generally more vigorous responding by that hemisphere.

As suggested in Ponton et al. (2009), whether a vMMN is obtained for speech stimuli could depend on stimulus kinematics. The current study took into account kinematics and the different deviation points across the different stimulus pairs. Inasmuch as the vMMN is expected to arise following deviation onset (Leitman et al., 2009), establishing the correct time point from which to measure the vMMN is critical. A method was devised here to establish the onset of stimulus deviation. The method was fairly gross, involving inspection of the video frames and measurement of the lip-opening area to align the stimuli within phoneme category and establish deviation across categories (Figure 2), but it resulted in good correspondence of the vMMNs latencies across stimuli and with previous positive reports (Ponton et al., 2009; Winkler et al., 2009).

The distributed dipole models of the standard stimuli here (Figures 4–6) suggest that the posterior temporal cortex responds to speech stimuli by 170–190 ms post-stimulus onset and continues to respond for ~200 ms. This interval is commensurate with the reliable vMMNs here (Table 2), which were measured using the electrode locations approximately over the posterior temporal response foci in the distributed dipole models. The results here are considered strong evidence that there is a posterior visual speech deviance response that is sensitive to consonant dissimilarity, but that detailed attention to stimulus attributes may be needed on the part of researchers in order to obtain it reliably.

### Hemispheric asymmetry of visual speech stimulus processing

Beyond demonstrating that visual speech deviance is responded to by high-level visual cortices, the current study focused on the hypothesis that the right and left posterior temporal cortices would demonstrate lateralized processing. The distributed dipole source models (Figures 4–6) show somewhat different areas of posterior temporal cortex to have been activated by each of the standard stimuli. In addition, during the first 400–500 ms post-stimulus onset, the activation appears to be greater for the right hemisphere.

There are published results that support functional anatomical asymmetry for processing non-speech face stimuli. For example, the right pSTS has been shown to be critically involved in processing eye gaze stimuli (Ethofer et al., 2011). In an ERP study alternating mouth open and mouth closed stimuli, the most prominent effect was a posterior negative potential around 170 ms which appeared to be larger on the right but was not reliably so (Puce et al., 2003). The researchers point out that the low spatial resolution with ERPs precludes the possibility of attributing their obtained effects exclusively to pSTS, because close cortical areas such as the human motion processing area (V5/MT) could also contribute to activation that appears to be localized to pSTS. Thus, although there is evidence in their study and here of different functional specialization across hemispheres, the indeterminacies with EEG source modeling preclude strong statements about the specific neuroanatomical regions activated within the posterior temporal cortices. However, an fMRI study (Bernstein et al., 2011), in which localizers were used did show that V5/MT was under-activated by visual speech in contrast with non-speech stimuli.

The left posterior temporal EOI deviance responses here are consistent with the temporal visual speech area (TVSA) reported by Bernstein et al. (2011) and are generally consistent with observations in other neuroimaging studies of lipreading (Calvert and Campbell, 2003; Paulesu et al., 2003; Skipper et al., 2005; Capek et al., 2008). The TVSA appears to be in the pathway that is also attributed with multisensory speech integration (Calvert, 2001; Nath and Beauchamp, 2011). The current results are consistent with the suggestion (Bernstein et al., 2011) that visual speech stimuli are extensively processed by the visual system prior to being mapped to higher-level speech representations, including semantic representations, in more anterior temporal cortices (Scott and Johnsrude, 2003; Hickok and Poeppel, 2007).

The right- vs. left-hemisphere vMMN results could be viewed as paradoxical under the assumption that sensitivity to speech stimulus deviation is evidence for specialization for speech. That is, the four vMMNs on the right might seem to afford more speech processing information than the two on the left. Here, the near deviant stimuli were discriminable as different patterns of speech gestures. But the obtained *d*′ discrimination measures that were ~1.4 for *near* contrasts are commensurate with previous results that showed the stimuli are not reliably labeled as different speech phonemes (Jiang et al., 2007a). Stimulus categorization involves generalization across small and/or irrelevant stimulus variation (Goldstone, 1994; Jiang et al., 2007b). Neural representations are the recipients of convergent and divergent connections, such that different lower-level representations can map to the same higher-level representation, and similar lower-level representations can map to different higher-level representations (Ahissar et al., 2008). Small stimulus differences that do not signal different phonemes could be mapped to the same representations on the left but mapped to different representations on the right (Figure 1).

The vMMNs on the left are explicitly not attributed to phoneme category representations but to the representation of the exogenous stimulus forms that are mapped to category representations, an organizational arrangement that is observed for non-speech visual object processing (Grill-Spector et al., 2006; Jiang et al., 2007b). This type of organization is also thought to be true for auditory speech processing, which is initiated at the cortical level with basic auditory features (e.g., frequencies, amplitudes) that are projected to exogenous phonetic stimulus forms, and then to higher-level phoneme, syllable, or lexical category representations (Binder et al., 2000; Scott et al., 2000; Eggermont, 2001; Scott, 2005; Hickok and Poeppel, 2007; Obleser and Eisner, 2009; May and Tiitinen, 2010; Näätänen et al., 2011).

Thus, the sensitivity of the left posterior temporal cortex to larger deviations only is expected for a lateralized language processing system that needs exogenous stimulus representations that can be reliably mapped to higher-level categories (Binder et al., 2000; Spitsyna et al., 2006). The deviation detection on the right could be more tightly integrated into a system responsive to social and affective signals (Puce et al., 2003), for which an inventory of categories such as phonemes that are combinatorically arranged is not required. For example, the right-hemisphere sensitivity to smaller stimulus deviations could be related to processing of emotion or visual attention stimuli (Puce et al., 1998, 2000, 2003; Wheaton et al., 2004; Thompson et al., 2007).

### Dissimilarity

Here, four vMMNs were sought in a design incorporating between- and within-consonant category stimuli and estimates of between-consonant category perceptual dissimilarity (Files and Bernstein, submitted; Jiang et al., 2007a). The perceptual dissimilarities were confirmed, and the vMMNs were consistent with the discrimination measures: Larger *d*′ was associated with larger vMMNs as predicted based on the expectation that the extent of neuronal representation overlap is related to the magnitude of the vMMN (Winkler and Czigler, 2012) (Figure 1). The direct comparison of the vMMN difference waves showed that, while holding stimulus constant (i.e., “zha”), the magnitude of the vMMN varied reliably with the context in which it was obtained. In the far (“fa”) context, the vMMN was larger than in the near (“ta”) context. To our knowledge, this is the first demonstration of predicted and reliable relative difference in the vMMN as a function of visual speech discriminability. This finding was also supported by the results for the other two stimuli, “ta” and “fa.”

These results converge with previous results on the relationship between visual speech discrimination and the physical visual stimuli. Jiang et al. (2007a) showed that the perceptual dissimilarity space obtained through multidimensional scaling of visual speech phoneme identification can be accounted for in terms of a physical (i.e., 3D optical) perceptually (linearly) warped multidimensional speech stimulus space. Files and Bernstein (in submission) followed up on those results and showed that the same dissimilarity space successfully predicts perceptual discrimination of the consonants. That is, the modeled perceptual dissimilarities based on perceptually warped stimulus differences predicted discrimination results and the deviance responses here.

The controlled dissimilarity factor in the current experiment afforded a unique approach to investigation of hemispheric specialization for visual speech processing. An alternate approach would be to compare ERPs obtained with speech vs. non-speech face gestures, as has been done in an fMRI experiment (Bernstein et al., 2011). However, that particular approach could introduce uncontrolled factors such as different salience of speech vs. non-speech stimuli. The current vMMN results also contribute a new insight about speech perception beyond that obtained within the Jiang et al. (2007a), and Files and Bernstein (in submission) perceptual studies. Specifically, the results here suggest that two types of representations can contribute to the perceptual discriminability of visual speech stimuli, speech consonant representations and face gesture representations.

### Mechanisms of the vMMN response

One of the goals of vMMN research, and MMN research more generally, has been to establish the mechanism/s that are responsible for the brain's response to stimulus deviance (Jaaskelainen et al., 2004; Näätänen et al., 2005, 2007; Kimura et al., 2009; May and Tiitinen, 2010). A main issue has been whether the cortical response to deviant stimuli is a so-called “higher-order memory-based process” or a neural adaptation effect (May and Tiitinen, 2010). The traditional paradigm for deriving the MMN (i.e., subtracting the ERP based on responses to standards from the ERP based on responses to deviants when deviant and standard are the same stimulus) was designed to show that the deviance response is a memory-based process. But the issue then arose whether the MMN is due entirely instead to refractoriness or adaptation of the same neuronal population activated by the same stimulus in its two different roles. The so-called “equiprobable paradigm” was designed to control for effects of refractoriness separate from deviance detection (Schroger and Wolff, 1996, 1997). The current study did not make use of the equiprobable paradigm, and we did not seek to address through our experimental design the question whether the deviance response is due to refractoriness/adaptation or a separate memory mechanism. We do think that our design rules out low-level stimulus effects and points to higher-level deviance detection responses at the level of speech processing.

The stimuli presented in the current vMMN experiment were not merely repetitions of the exact same stimulus. Deviants and standards were two different video tokens whose stimulus attributes differed (see Figure 2). These stimulus differences were such that it was necessary to devise a method to bring them into alignment with each other and to define deviations points, which were different depending on which vMMN was being analyzed. Furthermore, the stimuli were slightly jittered in position on the video monitor during presentation to defend additionally against low-level effects of stimulus repetition. Thus, the deviation detection at issue was relevant to consonant stimulus forms. We interpret the lateralization effects to be the result of the left hemisphere being more specialized for linguistically-relevant stimulus forms and the right hemisphere being more specialized for facial gestures that while not necessarily being discrete categories were nevertheless detected as different gestures (Puce et al., 1996). However, these results do not adjudicate between explanations that attempt to separate adaptation/refractoriness from an additional memory comparison process.

### vMMN to attended stimuli

The auditory MMN is known to be obtained both with and without attention (Näätänen et al., 1978, 2005, 2007). Similarly, the vMMN can be elicited in the absence of attention (Winkler et al., 2005; Czigler, 2007; Stefanics et al., 2011, 2012). Here, participants were required to attend to the stimuli and carry out a phoneme-level target detection task. Visual attention can result in attention-related ERP components in a similar latency range as the vMMN. A negativity on posterior lateral electrodes is commonly observed and is referred to as the *posterior N2, N2c*, or *selection negativity* (SN) (Folstein and Petten, 2008). However, the current results are not likely attributable to the SN, as the magnitude of the vMMN increased with perceptual dissimilarity of the standard from the deviant, whereas the SN is expected to increase with perceptual similarity of the deviant to a task-relevant target (Baas et al., 2002; Proverbio et al., 2009). Here, the *target* consonant was chosen to be equally dissimilar from both the *standard* and the *deviant* stimuli in a block, and this dissimilarity was similar across blocks. Therefore, differences in vMMN across syllables are unlikely attributable to the similarity of the *deviant* to the *target*: The task was constant in terms of the discriminability of the target, but the vMMNs varied in amplitude.

### No auditory MMN

Results of this study do not support the hypothesis that visual speech deviations are exogenously processed by the auditory cortex (Sams et al., 1991; Möttönen et al., 2002). This possibility received attention previously in the literature (e.g., Calvert et al., 1997; Bernstein et al., 2002; Pekkola et al., 2005). Seen vocalizations can modulate the response of auditory cortex (Möttönen et al., 2002; Pekkola et al., 2006; Saint-Amour et al., 2007), but the dipole source models of ERPs obtained with standard stimuli (Figures 4–6) do not show sources that can be attributed to the region of the primary auditory cortex. Nonetheless, the Fz and Cz ERPs obtained with standards and deviants were compared in part because of the possibility that an MMN reminiscent of an auditory MMN (Näätänen et al., 2007) might be obtained. Instead, a reliable positivity was found for the two *far* syllable contrasts. The timing of this positivity was similar to that of the vMMN observed on posterior temporal electrodes but was opposite in polarity. Similar positivities have been reported for other vMMN experiments and could reflect inversion of the posterior vMMN or some related but distinct component (Czigler et al., 2002, 2004).

### Summary and conclusions

Previous reports on the vMMN with visual speech stimuli were mixed, with relatively little evidence obtained for a visual deviation detection response. Here, the details of the visual stimuli were carefully observed for their deviations points. The possibility was taken into account that across hemispheres the two posterior temporal cortices represent speech stimuli differently. The left posterior temporal cortex, hypothesized to represent visual speech forms as input to a left-lateralized language processing system, was predicted to be responsive to perceptually large deviations between consonants. The right hemisphere, hypothesized to be sensitive to face and eye movements, was predicted to detect both perceptually large and small deviations between consonants. The predictions were shown to be correct. The vMMNs that were obtained for the perceptually *far* deviants were reliable bilaterally over posterior temporal cortices, but the vMMNs for the perceptually *near* deviants were reliably observed only over the right posterior temporal cortex. The results support a left-lateralized visual speech processing system.

### Conflict of interest statement

The authors declare that the research was conducted in the absence of any commercial or financial relationships that could be construed as a potential conflict of interest.

## Footnotes

^1^This approach does not account for different refractoriness or adaptation due to different probabilities of stimulus presentation (Schroger and Wolff, 1996; Czigler et al., 2002; Kimura et al., 2009). However, an additional set of control recordings would have been needed to take this into account, and here the focus was not on isolating a unique MMN component. Also, the design of the experiment would have been excessively long (see General Discussion).

^2^The alternate EOI clusters that were analyzed were: left (TP7 CP5 P7 P5), right (CP6 TP8 P6 P8); left (CP5 CP3 CP1 P7 P5 P3 P1 PO7 PO5 PO3 CB1) right (CP2 CP4 CP6 P2 P4 P6 P8 PO4 PO6 PO8 CB2); and left (TP7 CP5 CP3 CP1 P7 P5 P3 P1 PO7 PO5 PO3 CB1), right (CP2 CP4 CP6 TP8 P2 P4 P6 P8 PO4 PO6 PO8 CB2).

## Supplementary material

Figure S1 **(A)** ERP montage for “zha,” in the *far* context. Group mean ERPs for “zha” as *standard* in blocks with “fa” as *deviant*, and “zha” as *deviant* in blocks with “fa” as *standard*. **(B)** ERP montage for “zha,” in the *near* context. Group mean ERPs for “zha” as *standard* in blocks with “ta” as *deviant*, and “zha” as *deviant* in blocks with “ta” as *standard*. Each sub-axis shows the ERP on a different electrode, and the location of each axis maps to the location of that electrode on a head as seen from above, with the nose pointed up toward the top of the figure. The light green boxes show the electrodes of interest selected for subsequent vMMN analyses. Times shown are relative to deviation onset.

Figure S2 **(A)** ERP montage for “fa,” in the *far* context. Group mean ERPs for “fa” as *standard* in blocks with “zha” as *deviant* and “fa” as *deviant* in blocks with “zha” as a *standard*. **(B)** ERP montage for “ta,” in the *near* context. Group mean ERPs for “ta” as *standard* in blocks with “zha” as *deviant* and “ta” as *deviant* in blocks with “zha” as *standard*. The light green boxes show the electrodes of interest selected for subsequent vMMN analyses. Times shown are relative to deviation onset.

Figure S3 **Source images for “ta” *near* vMMN**. Images show the depth-weighted minimum norm estimate of dipole source strength constrained to the surface of the cortex using a boundary element forward model and a generic anatomical model at 20-ms intervals from 0 to 500 ms post-deviation onset. Images are thresholded at 20 pA·m. Foci of activity are scattered and transient, but focal activation occurs in fronto-central cortex throughout the time depicted, in right lateral occipital cortex from 0 to 40 ms, right posterior temporal cortex from 160 to 220 ms and 340 to 500 ms. Activation in the left hemisphere is scattered and transient throughout the time depicted.

Figure S4 **Source images for “fa” *far* vMMN**. Images show the depth-weighted minimum norm estimate of dipole source strength constrained to the surface of the cortex using a boundary element forward model and a generic anatomical model at 20-ms intervals from 0 to 500 ms post-deviation onset. Images are thresholded at 20 pA·m. Strong focal activity occurs in right lateral occipital cortex starting at ~260 ms, spreading into right posterior temporal cortex by 340 ms and expanding to include large swaths of posterior right cortex through the end of the temporal interval. In the left hemisphere, posterior temporal activity begins at ~280 ms and continuing through 400 ms at which time a more inferior focus in posterior/middle temporal cortex emerges and continues through the end of the temporal interval. Left fronto-central activity begins at ~300 ms and continues through to the end of the interval.

Figure S5 **Source images for “zha” *near* vMMN**. Images show the depth-weighted minimum norm estimate of dipole source strength constrained to the surface of the cortex using a boundary element forward model and a generic anatomical model at 20-ms intervals from 0 to 500 ms post-deviation onset. Images are thresholded at 20 pA·m. Strong activity in right posterior temporal/lateral occipital cortex begins at ~200 ms and proceeds through to 360 ms and then recurs from 440 ms to the end of the temporal interval. In the left hemisphere, activity is scattered and transient, but there are hotspots of activity in inferior frontal cortex from 200 to 240 ms, in fronto-central cortex from 320 to 420 ms and inferior posterior temporal cortex from 360 ms to the end of the interval depicted.

Figure S6 **Source images for “zha” *far* vMMN**. Images show the depth-weighted minimum norm estimate of dipole source strength constrained to the surface of the cortex using a boundary element forward model and a generic anatomical model at 20-ms intervals from 0 to 500 ms post-deviation onset. Images are thresholded at 20 pA·m. Focal activity in right posterior temporal cortex begins at 240 ms and continues through the end of the temporal interval, spreading to posterior inferior temporal and lateral occipital cortex at ~340 ms. Right fronto-lateral activity begins at 260 ms and continues through the end of the interval. Left fronto-central activity begins at 220 ms and continues through the end of the interval. Left posterior temporal activity occurs from 200 to 380 ms and in a slightly more inferior region from 460 ms to the end of the temporal interval.

## References

[B1] Ahissar M. Nahum M. Nelken I. Hochstein S. (2008). Reverse hierarchies and sensory learning. Philos. Trans. R. Soc. B 364, 285–299 10.1098/rstb.2008.0253 18986968 PMC2674477

[B2] Auer E. T. Jr. Bernstein L. E. (1997). Speechreading and the structure of the lexicon: computationally modeling the effects of reduced phonetic distinctiveness on lexical uniqueness. J. Acous. Soc. Am. 102, 3704–3710 10.1121/1.420402 9407662

[B3] Auer E. T. Jr. Bernstein L. E. (2007). Enhanced visual speech perception in individuals with early-onset hearing impairment. J. Speech Lang. Hear. Res. 50, 1157–1165 10.1044/1092-4388(2007/080) 17905902

[B4] Baas J. M. Kenemans J. L. Mangun G. R. (2002). Selective attention to spatial frequency: an ERP and source localization analysis. Clin. Neurophysiol. 113, 1840–1854 12417240 10.1016/s1388-2457(02)00269-9

[B5] Baillet S. Mosher J. C. Leahy R. M. (2001). Electromagnetic brain mapping. Signal Process. Mag. IEEE 18, 14–30 10.1109/79.962275

[B6] Bernstein L. E. (2012). Visual speech perception, in AudioVisual Speech Processing, eds Vatikiotis-Bateson E. Bailly G. Perrier P. (Cambridge: Cambridge University), 21–39 10.1017/CBO9780511843891.004

[B7] Bernstein L. E. Auer E. T. Jr. Moore J. K. Ponton C. W. Don M. (2002). Visual speech perception without primary auditory cortex activation. Neuroreport 13, 311–315 10.1097/00001756-200203040-00013 11930129

[B8] Bernstein L. E. Auer E. T. Jr. Wagner M. Ponton C. W. (2008). Spatiotemporal dynamics of audiovisual speech processing. Neuroimage 39, 423–435 10.1016/j.neuroimage.2007.08.035 17920933 PMC2185744

[B9] Bernstein L. E. Demorest M. E. Tucker P. E. (2000). Speech perception without hearing. Percep. Psychophys. 62, 233–252 10.3758/BF03205546 10723205

[B10] Bernstein L. E. Jiang J. Pantazis D. Lu Z. L. Joshi A. (2011). Visual phonetic processing localized using speech and nonspeech face gestures in video and point-light displays. Hum. Brain Mapp. 32, 1660–1676 10.1002/hbm.21139 20853377 PMC3120928

[B11] Besle J. Fort A. Delpuech C. Giard M.-H. (2004). Bimodal speech: early suppressive visual effects in human auditory cortex. Eur. J. Neurosci. 20, 2225–2234 10.1111/j.1460-9568.2004.03670.x 15450102 PMC1885424

[B12] Binder J. R. Frost J. A. Hammeke T. A. Bellgowan P. S. Springer J. A. Kaufman J. N. (2000). Human temporal lobe activation by speech and nonspeech sounds. Cereb. Cortex 10, 512–528 10.1093/cercor/10.5.512 10847601

[B13] Blair R. C. Karniski W. (1993). An alternative method for significance testing of waveform difference potentials. Psychophysiology 30, 518–524 10.1111/j.1469-8986.1993.tb02075.x 8416078

[B14] Calvert G. A. (2001). Crossmodal processing in the human brain: insights from functional neuroimaging studies. Cereb. Cortex 11, 1110–1123 10.1093/cercor/11.12.1110 11709482

[B15] Calvert G. A. Bullmore E. T. Brammer M. J. Campbell R. Williams S. C. McGuire P. K. (1997). Activation of auditory cortex during silent lipreading. Science 276, 593–596 10.1126/science.276.5312.593 9110978

[B16] Calvert G. A. Campbell R. (2003). Reading speech from still and moving faces: the neural substrates of visible speech. J. Cogn. Neurosci. 15, 57–70 10.1162/089892903321107828 12590843

[B17] Campbell R. (1986). The lateralization of lip-read sounds: a first look. Brain Cogn. 5, 1–21 10.1016/0278-2626(86)90059-X 3954902

[B18] Campbell R. (2011). Speechreading and the Bruce-Young model of face recognition: early findings and recent developments. Br. J. Psychol. 102, 704–710 10.1111/j.2044-8295.2011.02021.x 21988379

[B19] Campbell R. Landis T. Regard M. (1986). Face recognition and lipreading. A Neurological Dissociation. Brain 109(Pt 3), 509–521 10.1093/brain/109.3.509 3719288

[B20] Campbell R. Macsweeney M. Surguladze S. Calvert G. McGuire P. Suckling J. (2001). Cortical substrates for the perception of face actions: an fMRI study of the specificity of activation for seen speech and for meaningless lower-face acts (gurning). Cogn. Brain Res. 12, 233–243 10.1016/S0926-6410(01)00054-4 11587893

[B21] Capek C. M. Macsweeney M. Woll B. Waters D. McGuire P. K. David A. S. (2008). Cortical circuits for silent speechreading in deaf and hearing people. Neuropsychologia 46, 1233–1241 10.1016/j.neuropsychologia.2007.11.026 18249420 PMC2394569

[B22] Colin C. Radeau M. Soquet A. Deltenre P. (2004). Generalization of the generation of an MMN by illusory McGurk percepts: voiceless consonants. Clin. Neurophysiol. 115, 1989–2000 10.1016/j.clinph.2004.03.027 15294201

[B23] Colin C. Radeau M. Soquet A. Demolin D. Colin F. Deltenre P. (2002). Mismatch negativity evoked by the McGurk-MacDonald effect: a phonetic representation within short-term memory. Clin. Neurophysiol. 113, 495–506 10.1016/S1388-2457(02)00024-X 11955994

[B24] Czigler I. (2007). Visual mismatch negativity: violation of nonattended environmental regularities. J. Psychophysiol. 21, 224–230 10.1027/0269-8803.21.34.224

[B25] Czigler I. Balazs L. Pato L. G. (2004). Visual change detection: event-related potentials are dependent on stimulus location in humans. Neurosci. Lett. 364, 149–153 10.1016/j.neulet.2004.04.048 15196665

[B26] Czigler I. Balazs L. Winkler I. (2002). Memory-based detection of task-irrelevant visual changes. Psychophysiology 39, 869–873 10.1111/1469-8986.3960869 12462515

[B27] Dahaene-Lambertz G. (1997). Electrophysiological correlates of categorical phoneme perception in adults. Neuroreport 8, 919–924 10.1097/00001756-199703030-00021 9141065

[B28] Darvas F. Ermer J. J. Mosher J. C. Leahy R. M. (2006). Generic head models for atlas-based EEG source analysis. Hum. Brain Mapp. 27, 129–143 10.1002/hbm.20171 16037984 PMC6871464

[B29] Delorme A. Makeig S. (2004). EEGLAB: an open source toolbox for analysis of single-trial EEG dynamics including independent component analysis. J. Neurosci. Methods 134, 9–21 10.1016/j.jneumeth.2003.10.009 15102499

[B30] Edgington E. S. Onghena P. (2007). Randomization Tests. Boca Raton, FL: Chapman and Hall/CRC

[B31] Eggermont J. J. (2001). Between sound and perception: reviewing the search for a neural code. Hear. Res. 157, 1–42 10.1016/S0378-5955(01)00259-3 11470183

[B32] Ethofer T. Gschwind M. Vuilleumier P. (2011). Processing social aspects of human gaze: a combined fMRI-DTI study. Neuroimage 55, 411–419 10.1016/j.neuroimage.2010.11.033 21095230

[B33] Folstein J. R. Petten C. V. (2008). Influence of cognitive control and mismatch on the N2 component of the ERP: a review. Psychophysiology 45, 152–170 17850238 10.1111/j.1469-8986.2007.00602.x PMC2365910

[B34] Fuchs M. Wagner M. Köhler T. Wischmann H. A. (1999). Linear and nonlinear current density reconstructions. J. Clin. Neurophysiol. 16, 267–295 10.1097/00004691-199905000-00006 10426408

[B35] Goldstone R. L. (1994). Influences of categorization on perceptual discrimination. J. Exp. Psychol. Hum. Percep. Perform. 123, 178–200 10.1037/0096-3445.123.2.178 8014612

[B36] Gramfort A. Papadopoulo T. Olivi E. Clerc M. (2010). OpenMEEG: opensource software for quasistatic bioelectromagnetics. Biomed. Eng. Online 9:45 10.1186/1475-925X-9-45 20819204 PMC2949879

[B37] Green D. M. Swets J. A. (1966). Signal Detection Theory And Psychophysics. New York, NY: Wiley

[B38] Grill-Spector K. Henson R. Martin A. (2006). Repetition and the brain: neural models of stimulus-specific effects. Trends Cogn. Sci. 10, 14–23 10.1016/j.tics.2005.11.006 16321563

[B39] Grill-Spector K. Kourtzi Z. Kanwisher N. (2001). The lateral occipital complex and its role in object recognition. Vision Res. 41, 1409–1422 10.1016/S0042-6989(01)00073-6 11322983

[B40] Groppe D. M. Urbach T. P. Kutas M. (2011). Mass univariate analysis of event-related brain potentials/fields I: a critical tutorial review. Psychophysiology 48, 1711–1725 10.1111/j.1469-8986.2011.01273.x 21895683 PMC4060794

[B41] Guthrie D. Buchwald J. S. (1991). Significance testing of difference potentials. Psychophysiology 28, 240–244 10.1111/j.1469-8986.1991.tb00417.x 1946890

[B42] Hickok G. Poeppel D. (2007). The cortical organization of speech processing. Nat. Rev. Neurosci. 8, 393–402 10.1038/nrn2113 17431404

[B43] Jaaskelainen I. P. Ahveninen J. Bonmassar G. Dale A. M. Ilmoniemi R. J. Levanen S. (2004). Human posterior auditory cortex gates novel sounds to consciousness. Proc. Natl. Acad. Sci. U.S.A. 101, 6809–6814 10.1073/pnas.0303760101 15096618 PMC404127

[B44] Jesse A. Massaro D. W. (2010). The temporal distribution of information in audiovisual spoken-word identification. Attent. Percept. Psychophys. 72, 209–225 10.3758/APP.72.1.209 20045890

[B45] Jiang J. Alwan A. Keating P. Auer E. T. Jr. Bernstein L. E. (2002). On the relationship between face movements, tongue movements, and speech acoustics. EURASIP J. Appl. Signal Process. Spec. Issue Jt AudioVis. Speech Process. 2002, 1174–1188 10.1155/S1110865702206046

[B46] Jiang J. Auer E. T. Jr. Alwan A. Keating P. A. Bernstein L. E. (2007a). Similarity structure in visual speech perception and optical phonetic signals. Percep. Psychophys. 69, 1070–1083 10.3758/BF03193945 18038946

[B47] Jiang X. Bradley E. Rini R. A. Zeffiro T. Vanmeter J. Riesenhuber M. (2007b). Categorization training results in shape- and category-selective human neural plasticity. Neuron 53, 891–903 10.1016/j.neuron.2007.02.015 17359923 PMC1989663

[B48] Kanwisher N. McDermott J. Chun M. M. (1997). The fusiform face area: a module in human extrastriate cortex specialized for face perception. J. Neurosci. 17, 4302–4311 9151747 10.1523/JNEUROSCI.17-11-04302.1997 PMC6573547

[B49] Kecskes-Kovacs K. Sulykos I. Czigler I. (2013). Visual mismatch negativity is sensitive to symmetry as a perceptual category. Eur. J. Neurosci. 37, 662–667 10.1111/ejn.12061 23167956

[B50] Kimura M. Katayama J. Ohira H. Schroger E. (2009). Visual mismatch negativity: new evidence from the equiprobable paradigm. Psychophysiology 46, 402–409 10.1111/j.1469-8986.2008.00767.x 19207207

[B51] Kimura M. Schroger E. Czigler I. (2011). Visual mismatch negativity and its importance in visual cognitive sciences. Neuroreport 22, 669–673 10.1097/WNR.0b013e32834973ba 21878790

[B52] Kriegeskorte N. Simmons W. K. Bellgowan P. S. Baker C. I. (2009). Circular analysis in systems neuroscience: the dangers of double dipping. Nat. Neurosci. 12, 535–540 10.1038/nn.2303 19396166 PMC2841687

[B53] Kujala T. Tervaniemi M. Schroger E. (2007). The mismatch negativity in cognitive and clinical neuroscience: theoretical and methodological considerations. Biol. Psychol. 74, 1–19 10.1016/j.biopsycho.2006.06.001 16844278

[B54] Lehmann D. Skrandies W. (1980). Reference-free identification of components of checkerboard-evoked multichannel potential fields. Electroencephalogr. Clin. Neurophysiol. 48, 609–621 10.1016/0013-4694(80)90419-8 6155251

[B55] Leitman D. I. Sehatpour P. Shpaner M. Foxe J. J. Javitt D. C. (2009). Mismatch negativity to tonal contours suggests preattentive perception of prosodic content. Brain Imaging Behav. 3, 284–291 10.1007/s11682-009-9070-7 22005991 PMC3202295

[B56] Li X. Lu Y. Sun G. Gao L. Zhao L. (2012). Visual mismatch negativity elicited by facial expressions: new evidence from the equiprobable paradigm. Behav. Brain Funct. 8, 7 22300600 10.1186/1744-9081-8-7 PMC3292984

[B57] Macmillan N. A. Creelman C. D. (1991). Detection Theory: a User's Guide. Cambridge, UK; New York, NY: Cambridge University Press

[B58] May P. J. C. Tiitinen H. (2010). Mismatch negativity (MMN), the deviance-elicited auditory deflection, explained. Psychophysiology 47, 66–122 10.1111/j.1469-8986.2009.00856.x 19686538

[B59] Michel C. M. Murray M. M. Lantz G. Gonzalez S. Spinelli L. De Peralta R. G. (2004). EEG source imaging. Clin. Neurophys. 115, 2195–2222 10.1016/j.clinph.2004.06.001 15351361

[B60] Miki K. Watanabe S. Kakigi R. Puce A. (2004). Magnetoencephalographic study of occipitotemporal activity elicited by viewing mouth movements. Clin. Neurophysiol. 115, 1559–1574 10.1016/j.clinph.2004.02.013 15203057

[B61] Möttönen R. Krause C. M. Tiippana K. Sams M. (2002). Processing of changes in visual speech in the human auditory cortex. Cogn. Brain Res. 13, 417–425 10.1016/S0926-6410(02)00053-8 11919005

[B62] Näätänen R. Gaillard A. W. K. Mäntysalo S. (1978). Early selective-attention effect on evoked potential reinterpreted. Acta Psychol. 42, 313–329 10.1016/0001-6918(78)90006-9 685709

[B63] Näätänen R. Jacobsen T. Winkler I. (2005). Memory-based or afferent processes in mismatch negativity (MMN): a review of the evidence. Psychophysiology 42, 25–32 10.1111/j.1469-8986.2005.00256.x 15720578

[B64] Näätänen R. Kujala T. Winkler I. (2011). Auditory processing that leads to conscious perception: a unique window to central auditory processing opened by the mismatch negativity and related responses. Psychophysiology 48, 4–22 10.1111/j.1469-8986.2010.01114.x 20880261

[B65] Näätänen R. Paavilainen P. Rinne T. Alho K. (2007). The mismatch negativity (MMN) in basic research of central auditory processing: a review. Clin. Neurophysiol. 118, 2544–2590 10.1016/j.clinph.2007.04.026 17931964

[B66] Nath A. R. Beauchamp M. S. (2011). Dynamic changes in superior temporal sulcus connectivity during perception of noisy audiovisual speech. J. Neurosci. 31, 1704–1714 10.1523/JNEUROSCI.4853-10.2011 21289179 PMC3050590

[B67] Nath A. R. Beauchamp M. S. (2012). A neural basis for interindividual differences in the McGurk effect, a multisensory speech illusion. Neuroimage 59, 781–787 10.1016/j.neuroimage.2011.07.024 21787869 PMC3196040

[B68] Nichols T. E. Holmes A. P. (2002). Nonparametric permutation tests for functional neuroimaging: a primer with examples. Hum. Brain Mapp. 15, 1–25 10.1002/hbm.1058 11747097 PMC6871862

[B69] Nousak J. M. Deacon D. Ritter W. Vaughan H. G. Jr. (1996). Storage of information in transient auditory memory. Brain Res. Cogn. Brain Res. 4, 305–317 10.1016/S0926-6410(96)00068-7 8957572

[B70] Obleser J. Eisner F. (2009). Pre-lexical abstraction of speech in the auditory cortex. Trends Cogn. Sci. 31, 14–19 10.1016/j.tics.2008.09.005 19070534

[B71] Oldfield R. C. (1971). The assessment and analysis of handedness: the Edinburgh inventory. Neuropsychologia 9, 97–113 10.1016/0028-3932(71)90067-4 5146491

[B72] Paulesu E. Perani D. Blasi V. Silani G. Borghese N. A. De Giovanni U. (2003). A functional-anatomical model for lipreading. J. Neurophysiol. 90, 2005–2013 10.1152/jn.00926.2002 12750414

[B73] Pazo-Alvarez P. Amenedo E. Cadaveira F. (2004). Automatic detection of motion direction changes in the human brain. Eur. J. Neurosci. 19, 1978–1986 10.1111/j.1460-9568.2004.03273.x 15078572

[B74] Pazo-Alvarez P. Cadaveira F. Amenedo E. (2003). MMN in the visual modality: a review. Biol. Psychol. 63, 199–236 10.1016/S0301-0511(03)00049-8 12853168

[B75] Pekkola J. Ojanen V. Autti T. Jaaskelainen I. P. Mottonen R. Sams M. (2006). Attention to visual speech gestures enhances hemodynamic activity in the left planum temporale. Hum. Brain Mapp. 27, 471–477 10.1002/hbm.20190 16161166 PMC6871389

[B76] Pekkola J. Ojanen V. Autti T. Jaaskelainen I. P. Mottonen R. Tarkiainen A. (2005). Primary auditory cortex activation by visual speech: an fMRI study at 3 T. Neuroreport 16, 125–128 10.1097/00001756-200502080-00010 15671860

[B77] Picton T. W. Bentin S. Berg P. Donchin E. Hillyard S. A. Johnson R. (2000). Guidelines for using human event-related potentials to study cognition: recording standards and publication criteria. Psychophysiology 37, 127–152 10.1111/1469-8986.3720127 10731765

[B78] Ponton C. W. Bernstein L. E. Auer E. T. Jr. (2009). Mismatch negativity with visual-only and audiovisual speech. Brain Topogr. 21, 207–215 10.1007/s10548-009-0094-5 19404730 PMC2708318

[B79] Ponton C. W. Don M. Eggermont J. J. Kwong B. (1997). Integrated mismatch negativity (MMNi): a noise-free representation of evoked responses allowing single-point distribution-free statistical tests. Electroencephalogr. Clin. Neurophysiol. 104, 143–150 10.1016/S0168-5597(97)96104-9 9146480

[B80] Proverbio A. M. Del Zotto M. Crotti N. Zani A. (2009). A no-go related prefrontal negativity larger to irrelevant stimuli that are difficult to suppress. Behav. Brain Funct. 5, 25 19555496 10.1186/1744-9081-5-25 PMC2708178

[B81] Puce A. Allison T. Asgari M. Gore J. C. McCarthy G. (1996). Differential sensitivity of human visual cortex to faces, letterstrings, and textures: a functional magnetic resonance imaging study. J. Neurosci. 16, 5205–5215 8756449 10.1523/JNEUROSCI.16-16-05205.1996 PMC6579313

[B82] Puce A. Allison T. Bentin S. Gore J. C. McCarthy G. (1998). Temporal cortex activation in humans viewing eye and mouth movements. J. Neurosci. 18, 2188–2199 9482803 10.1523/JNEUROSCI.18-06-02188.1998 PMC6792917

[B83] Puce A. Epling J. A. Thompson J. C. Carrick O. K. (2007). Neural responses elicited to face motion and vocalization pairings. Neuropsychologia 45, 93–106 10.1016/j.neuropsychologia.2006.04.017 16766000 PMC2785010

[B84] Puce A. Smith A. Allison T. (2000). ERPs evoked by viewing facial movements. Cogn. Neuropsychol. 17, 221–239 10.1080/026432900380580 20945181

[B85] Puce A. Syngeniotis A. Thompson J. C. Abbott D. F. Wheaton K. J. Castiello U. (2003). The human temporal lobe integrates facial form and motion: evidence from fMRI and ERP studies. Neuroimage 19, 861–869 10.1016/S1053-8119(03)00189-7 12880814

[B86] Saint-Amour D. Sanctis P. D. Molholm S. Ritter W. Foxe J. J. (2007). Seeing voices: high-density electrical mapping and source-analysis of the multisensory mismatch negativity evoked during the McGurk illusion. Neuropsychologia 45, 587–597 10.1016/j.neuropsychologia.2006.03.036 16757004 PMC1705816

[B87] Sams M. Alho K. Naatanen R. (1984). Short-term habituation and dishabituation of the mismatch negativity of the ERP. Psychophysiology 21, 434–441 10.1111/j.1469-8986.1984.tb00223.x 6463176

[B88] Sams M. Aulanko R. Hämäläinen M. Hari R. Lounasmaa O. V. Lu S. T. (1991). Seeing speech: visual information from lip movements modifies activity in the human auditory cortex. Neurosci. Lett. 127, 141–145 10.1016/0304-3940(91)90914-F 1881611

[B89] Schroger E. Wolff C. (1996). Mismatch response of the human brain to changes in sound location. Neuroreport 7, 3005–3008 10.1097/00001756-199611250-00041 9116228

[B90] Schroger E. Wolff C. (1997). Fast preattentive processing of location: a functional basis for selective listening in humans. Neurosci. Lett. 232, 5–8 10.1016/S0304-3940(97)00561-2 9292878

[B91] Scott S. K. (2005). Auditory processing–speech, space and auditory objects. Curr. Opin. Neurobiol. 15, 197–201 10.1016/j.conb.2005.03.009 15831402

[B92] Scott S. K. Blank C. C. Rosen S. Wise R. J. (2000). Identification of a pathway for intelligible speech in the left temporal lobe. Brain 123(Pt 12), 2400–2406 10.1093/brain/123.12.2400 11099443 PMC5630088

[B93] Scott S. K. Johnsrude I. S. (2003). The neuroanatomical and functional organization of speech perception. Trends Neurosci. 26, 100–107 10.1016/S0166-2236(02)00037-1 12536133

[B94] Semlitsch H. V. Anderer P. Schuster P. Presslich O. (1986). A solution for reliable and valid reduction of ocular artifacts, applied to the P300 ERP. Psychophysiology 23, 695–703 10.1111/j.1469-8986.1986.tb00696.x 3823345

[B95] Skipper J. I. Nusbaum H. C. Small S. L. (2005). Listening to talking faces: motor cortical activation during speech perception. Neuroimage 25, 76–89 10.1016/j.neuroimage.2004.11.006 15734345

[B96] Skrandies W. (1990). Global field power and topographic similarity. Brain Topogr. 3, 137–141 10.1007/BF01128870 2094301

[B97] Spitsyna G. Warren J. E. Scott S. K. Turkheimer F. E. Wise R. J. (2006). Converging language streams in the human temporal lobe. J. Neurosci. 26, 7328–7336 10.1523/JNEUROSCI.0559-06.2006 16837579 PMC6674192

[B98] Stefanics G. Csukly G. Komlosi S. Czobor P. Czigler I. (2012). Processing of unattended facial emotions: a visual mismatch negativity study. Neuroimage 59, 3042–3049 10.1016/j.neuroimage.2011.10.041 22037000

[B99] Stefanics G. Kimura M. Czigler I. (2011). Visual mismatch negativity reveals automatic detection of sequential regularity violation. Front. Hum. Neurosci. 5:46 10.3389/fnhum.2011.00046 21629766 PMC3099311

[B100] Tadel F. Baillet S. Mosher J. C. Pantazis D. Leahy R. M. (2011). Brainstorm: a user-friendly application for MEG/EEG analysis. Comput. Intell. Neurosci. 2011, 879716 10.1155/2011/879716 21584256 PMC3090754

[B101] Thompson J. C. Hardee J. E. Panayiotou A. Crewther D. Puce A. (2007). Common and distinct brain activation to viewing dynamic sequences of face and hand movements. Neuroimage 37, 966–973 10.1016/j.neuroimage.2007.05.058 17616403

[B102] Wheaton K. J. Thompson J. C. Syngeniotis A. Abbott D. F. Puce A. (2004). Viewing the motion of human body parts activates different regions of premotor, temporal, and parietal cortex. Neuroimage 22, 277–288 10.1016/j.neuroimage.2003.12.043 15110018

[B103] Winkler I. Czigler I. (2012). Evidence from auditory and visual event-related potential (ERP) studies of deviance detection (MMN and vMMN) linking predictive coding theories and perceptual object representations. Int. J. Psychophysiol. 83, 132–143 10.1016/j.ijpsycho.2011.10.001 22047947

[B104] Winkler I. Czigler I. Sussman E. Horváth J. Balazs L. (2005). Preattentive binding of auditory and visual stimulus features. J. Cogn. Neurosci. 17, 320–339 10.1162/0898929053124866 15811243

[B105] Winkler I. Horváth J. Weisz J. Trejo L. J. (2009). Deviance detection in congruent audiovisual speech: evidence for implicit integrated audiovisual memory representations. Biol. Psychol. 82, 281–292 10.1016/j.biopsycho.2009.08.011 19733617

[B106] Yehia H. Rubin P. Vatikiotis-Bateson E. (1998). Quantitative association of vocal-tract and facial behavior. Speech Commun. 26, 23–43 10.1016/S0167-6393(98)00048-X

