# Supplementary figures and images for: The visual mismatch negativity elicited with visual speech stimuli
Source: Front Hum Neurosci. 2013 Jul 16;7:371. doi: 10.3389/fnhum.2013.00371 (PMC3712324; doi:10.3389/fnhum.2013.00371)

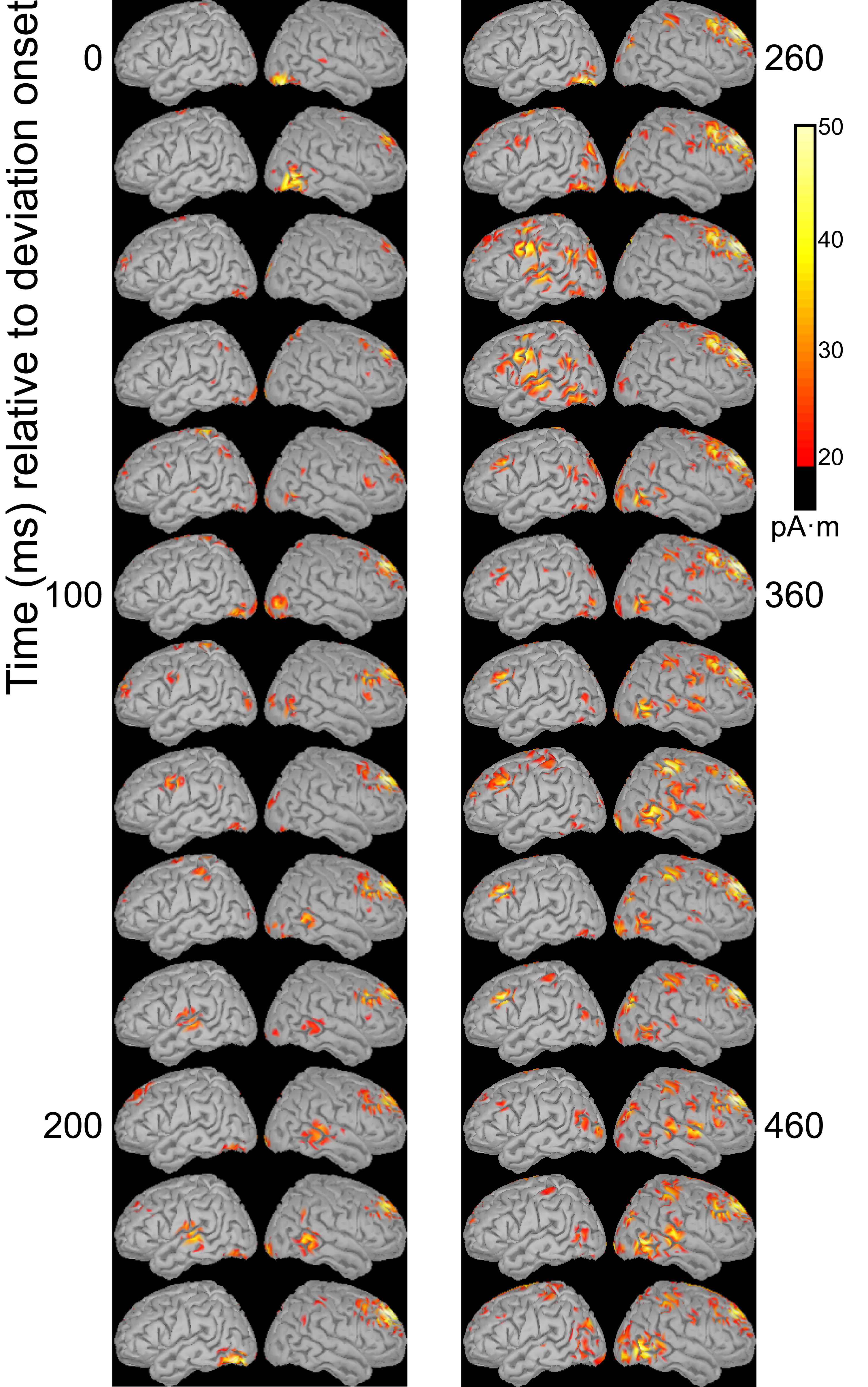

Supplement: Figure S1 — (A) ERP montage for “zha,” in the far context. Group mean ERPs for “zha” as standard in blocks with “fa” as deviant, and “zha” as deviant in blocks with “fa” as standard. (B) ERP montage for “zha,” in the near context. Group mean ERPs for “zha” as standard in blocks with “ta” as deviant, and “zha” as deviant in blocks with “ta” as standard. Each sub-axis shows the ERP on a different electrode, and the location of each axis maps to the location of that electrode on a head as seen from above, with the nose pointed up toward the top of the figure. The light green boxes show the electrodes of interest selected for subsequent vMMN analyses. Times shown are relative to deviation onset. [file 47680_Bernstein_Presentation1.ZIP › 47680_Bernstein_Figure_S3.TIF]

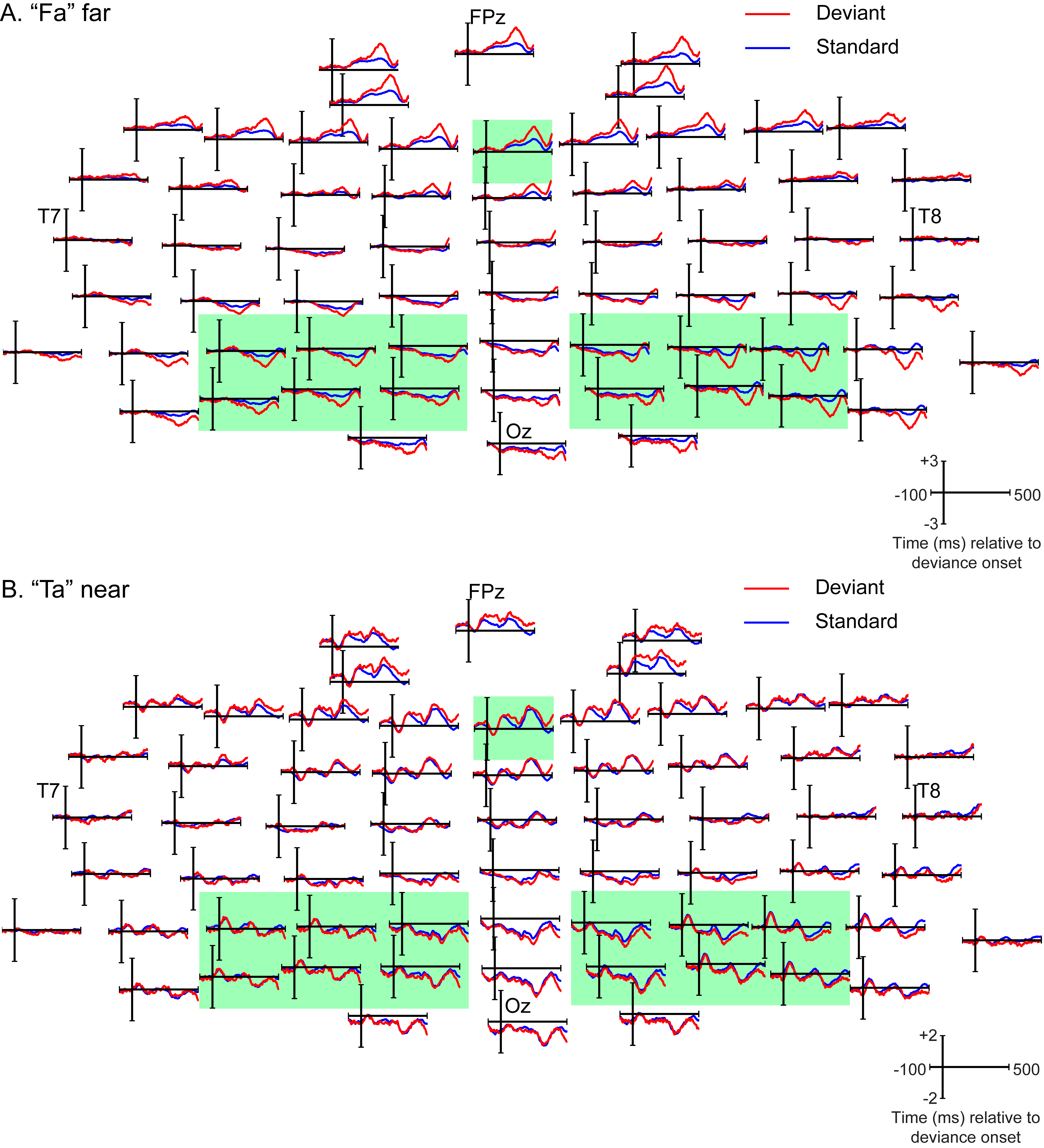

Supplement: Figure S1 — (A) ERP montage for “zha,” in the far context. Group mean ERPs for “zha” as standard in blocks with “fa” as deviant, and “zha” as deviant in blocks with “fa” as standard. (B) ERP montage for “zha,” in the near context. Group mean ERPs for “zha” as standard in blocks with “ta” as deviant, and “zha” as deviant in blocks with “ta” as standard. Each sub-axis shows the ERP on a different electrode, and the location of each axis maps to the location of that electrode on a head as seen from above, with the nose pointed up toward the top of the figure. The light green boxes show the electrodes of interest selected for subsequent vMMN analyses. Times shown are relative to deviation onset. [file 47680_Bernstein_Presentation1.ZIP › 47680_Bernstein_Figure_S2.TIF]

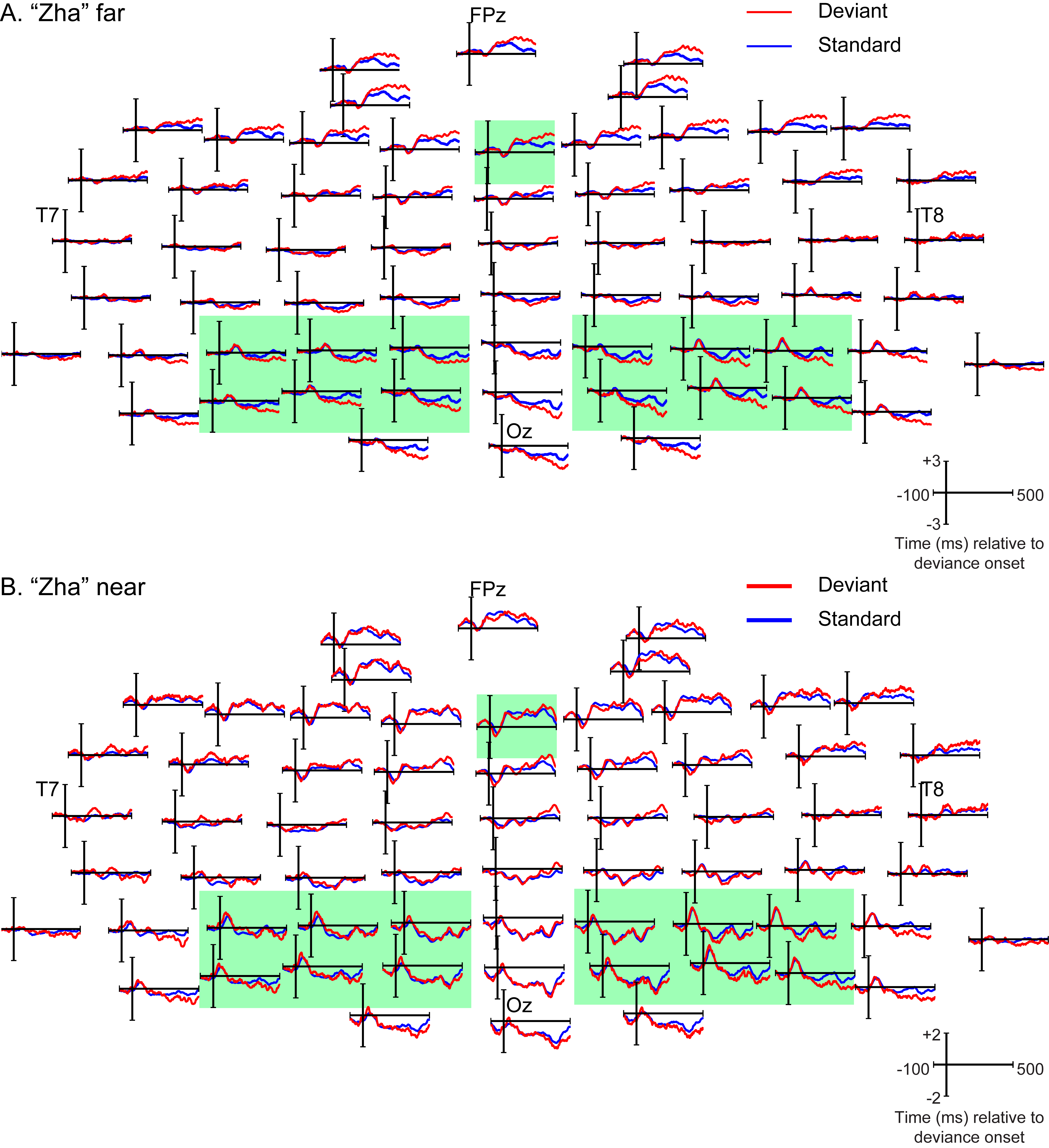

Supplement: Figure S1 — (A) ERP montage for “zha,” in the far context. Group mean ERPs for “zha” as standard in blocks with “fa” as deviant, and “zha” as deviant in blocks with “fa” as standard. (B) ERP montage for “zha,” in the near context. Group mean ERPs for “zha” as standard in blocks with “ta” as deviant, and “zha” as deviant in blocks with “ta” as standard. Each sub-axis shows the ERP on a different electrode, and the location of each axis maps to the location of that electrode on a head as seen from above, with the nose pointed up toward the top of the figure. The light green boxes show the electrodes of interest selected for subsequent vMMN analyses. Times shown are relative to deviation onset. [file 47680_Bernstein_Presentation1.ZIP › 47680_Bernstein_Figure_S1.TIF]

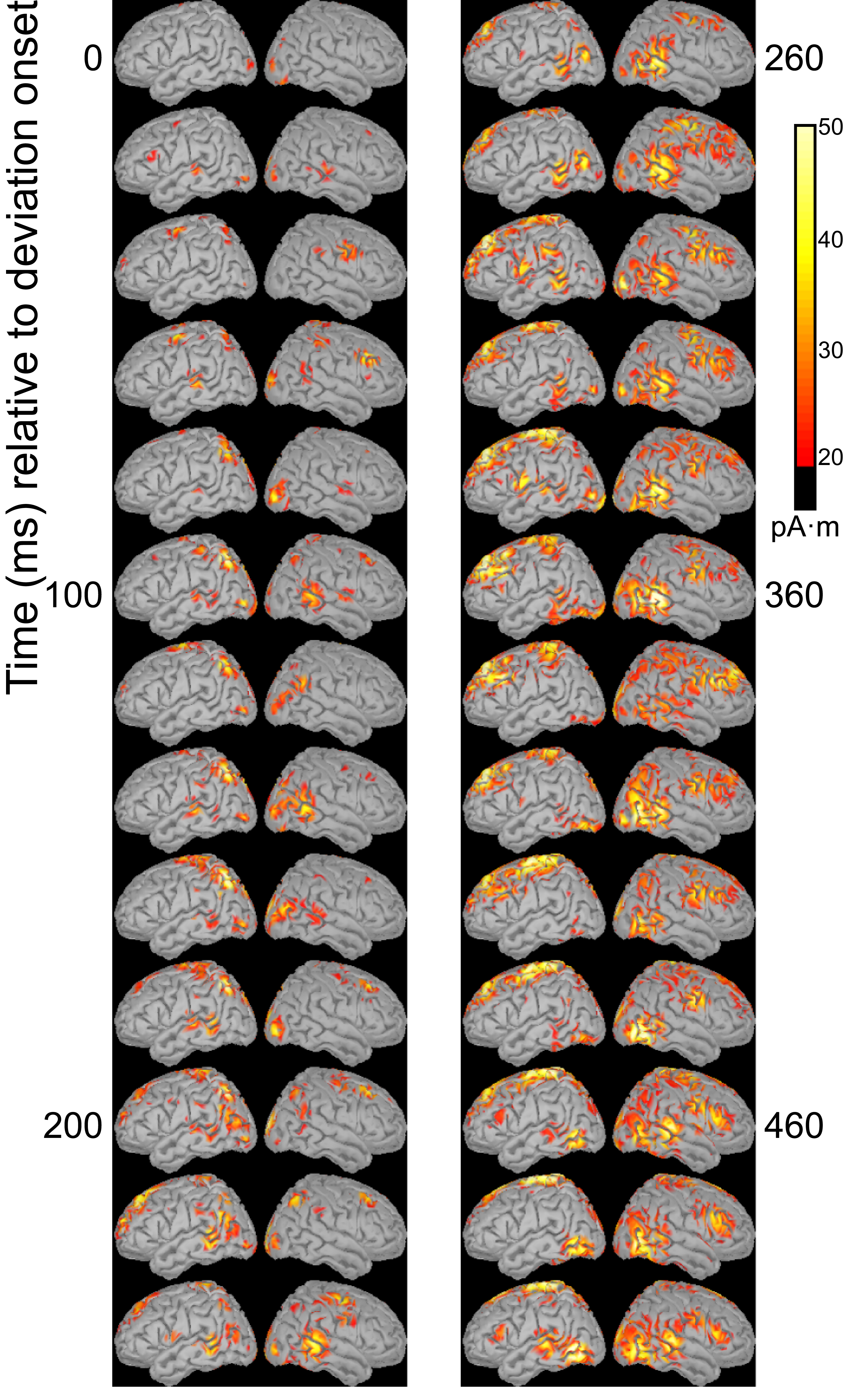

Supplement: Figure S1 — (A) ERP montage for “zha,” in the far context. Group mean ERPs for “zha” as standard in blocks with “fa” as deviant, and “zha” as deviant in blocks with “fa” as standard. (B) ERP montage for “zha,” in the near context. Group mean ERPs for “zha” as standard in blocks with “ta” as deviant, and “zha” as deviant in blocks with “ta” as standard. Each sub-axis shows the ERP on a different electrode, and the location of each axis maps to the location of that electrode on a head as seen from above, with the nose pointed up toward the top of the figure. The light green boxes show the electrodes of interest selected for subsequent vMMN analyses. Times shown are relative to deviation onset. [file 47680_Bernstein_Presentation1.ZIP › 47680_Bernstein_Figure_S6.TIF]

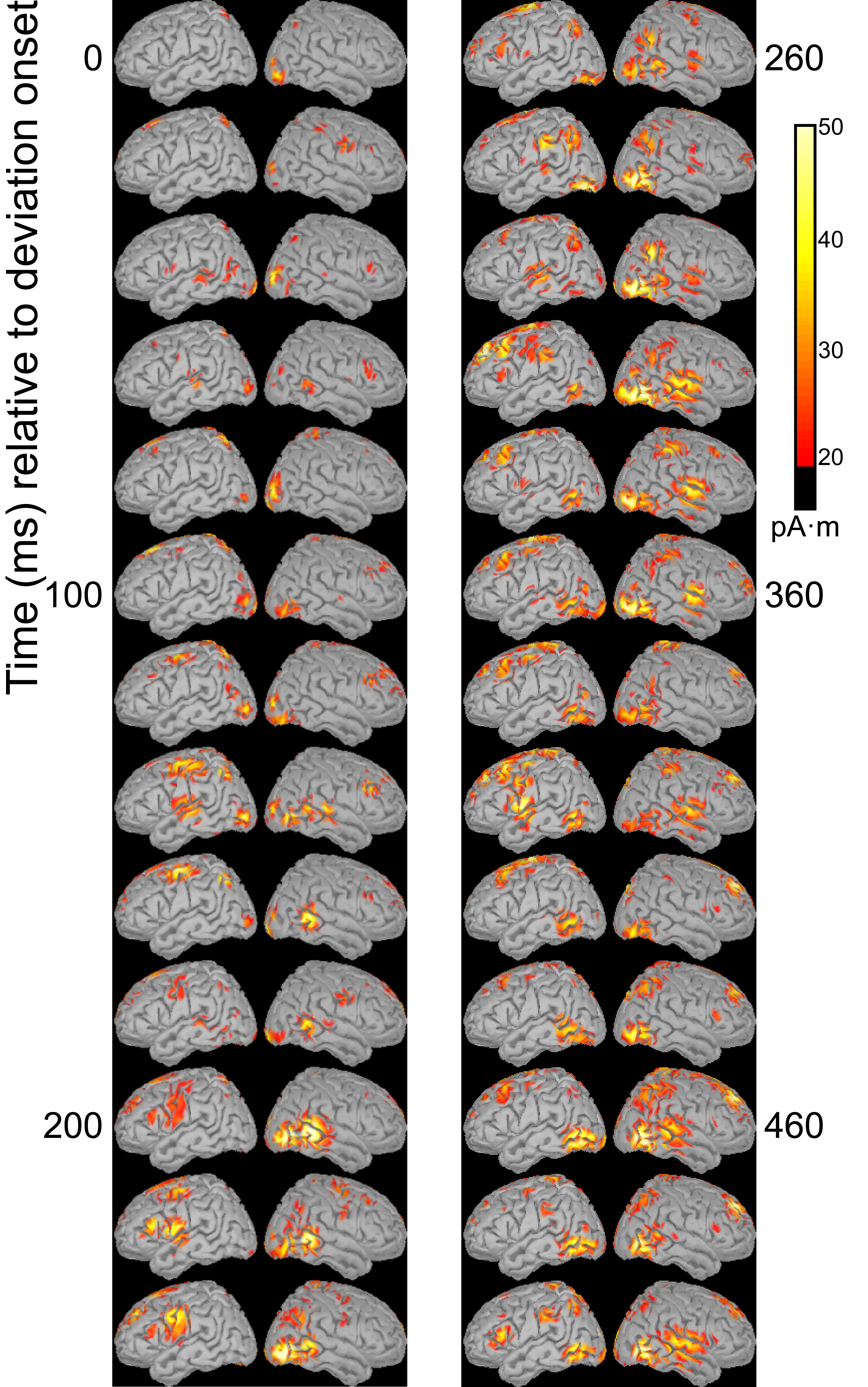

Supplement: Figure S1 — (A) ERP montage for “zha,” in the far context. Group mean ERPs for “zha” as standard in blocks with “fa” as deviant, and “zha” as deviant in blocks with “fa” as standard. (B) ERP montage for “zha,” in the near context. Group mean ERPs for “zha” as standard in blocks with “ta” as deviant, and “zha” as deviant in blocks with “ta” as standard. Each sub-axis shows the ERP on a different electrode, and the location of each axis maps to the location of that electrode on a head as seen from above, with the nose pointed up toward the top of the figure. The light green boxes show the electrodes of interest selected for subsequent vMMN analyses. Times shown are relative to deviation onset. [file 47680_Bernstein_Presentation1.ZIP › 47680_Bernstein_Figure_S5.TIF]

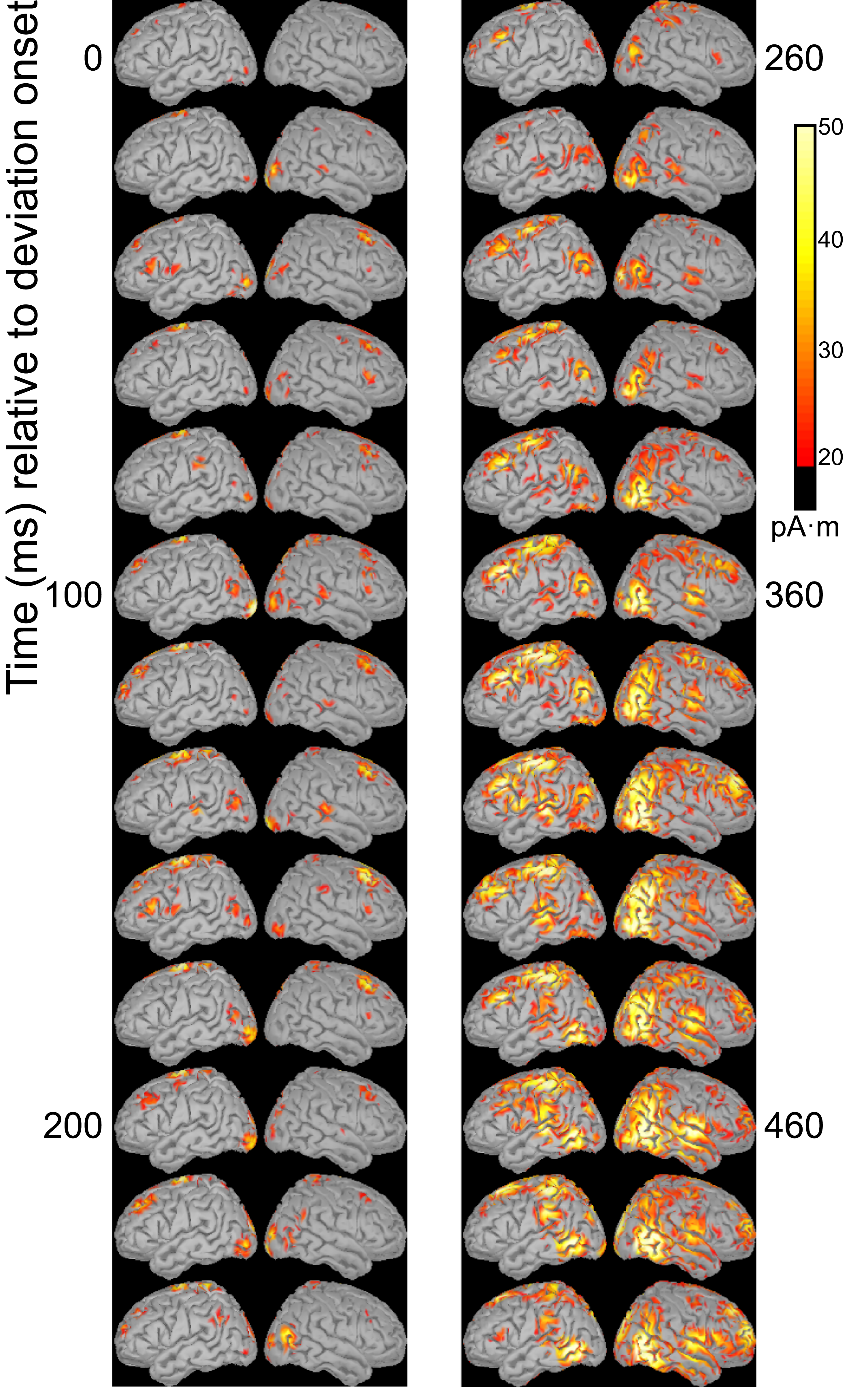

Supplement: Figure S1 — (A) ERP montage for “zha,” in the far context. Group mean ERPs for “zha” as standard in blocks with “fa” as deviant, and “zha” as deviant in blocks with “fa” as standard. (B) ERP montage for “zha,” in the near context. Group mean ERPs for “zha” as standard in blocks with “ta” as deviant, and “zha” as deviant in blocks with “ta” as standard. Each sub-axis shows the ERP on a different electrode, and the location of each axis maps to the location of that electrode on a head as seen from above, with the nose pointed up toward the top of the figure. The light green boxes show the electrodes of interest selected for subsequent vMMN analyses. Times shown are relative to deviation onset. [file 47680_Bernstein_Presentation1.ZIP › 47680_Bernstein_Figure_S4.TIF]
